# Future Transboundary Water Stress and Its Drivers Under Climate Change: A Global Study

**DOI:** 10.1029/2019EF001321

**Published:** 2020-06-29

**Authors:** Hafsa Ahmed Munia, Joseph H. A. Guillaume, Yoshihide Wada, Ted Veldkamp, Vili Virkki, Matti Kummu

**Affiliations:** ^1^ Water and Development Research Group Aalto University Espoo Finland; ^2^ The Fenner School of Environment & Society The Australian National University Canberra Australian Capital Territory Australia; ^3^ International Institute for Applied Systems Analysis Laxenburg Austria; ^4^ Department of Physical Geography, Faculty of Geosciences Utrecht University Utrecht The Netherlands; ^5^ Institute for Environmental Studies (IVM) Vrije Universiteit Amsterdam Amsterdam The Netherlands

**Keywords:** transboundary river, climate change, representative concentration pathways, shared socioeconomic pathways, water stress

## Abstract

Various transboundary river basins are facing increased pressure on water resources in near future. However, little is known ab out the future drivers globally, namely, changes in natural local runoff and natural inflows from upstream parts of a basin, as well as local and upstream water consumption. Here we use an ensemble of four global hydrological models forced by five global climate models and the latest greenhouse‐gas concentration (RCP) and socioeconomic pathway (SSP) scenarios to assess the impact of these drivers on transboundary water stress in the past and future. Our results show that population under water stress is expected to increase by 50% under a low population growth and emissions scenario (SSP1‐RCP2.6) and double under a high population growth and emission scenario (SSP3‐RCP6.0), compared to the year 2010. As changes in water availability have a smaller effect when water is not yet scarce, changes in water stress globally are dominated by local water consumption—managing local demand is thus necessary in order to avoid future stress. Focusing then on the role of upstream changes, we identified upstream availability (i.e., less natural runoff or increased water consumption) as the dominant driver of changes in net water availability in most downstream areas. Moreover, an increased number of people will be living in areas dependent on upstream originating water in 2050. International water treaties and management will therefore have an increasingly crucial role in these hot spot regions to ensure fair management of transboundary water resources.

## Key Points

- Water stress will mostly intensify in areas already under stress in all future scenarios for most transboundary river basins
- Changes in water stress are dominated by local water consumption—managing local demand is thus a key strategy to alleviate future stress
- Less natural runoff or increased water consumption in upstream areas is the dominant driver of changes in downstream net water availability

## 1 Introduction

Climate change has been identified as a potential impediment to effective long‐range policies and management of water resources (Draper & Kundell, 2007). About one third to half of the global population is currently experiencing physical water scarcity (Hanasaki et al., 2013; Kummu et al., 2010; Mekonnen & Hoekstra, 2016; Schewe et al., 2014; Vörösmarty et al., 2000). While climate change poses an additional threat to already stressed water resources by adding uncertainty, for example, regarding changes in temperature and precipitation (Schewe et al., 2014), a number of socioeconomic drivers also affect water resources and water stress (Alcamo et al., 2007; Arnell, 2004; Arnell & Lloyd‐Hughes, 2014; Veldkamp et al., 2017). Incorporating the evaluation of these socioeconomic pressures is thus very important while assessing the impact of climate change in future water resources, particularly when discussing adaptation plans for water management (Kiguchi et al., 2015; Kundzewicz et al., 2008). To tackle this issue, an international network of climate‐impact modelers works to provide a wide‐ranging and internally consistent picture of the world under different socioeconomic and concentration scenarios and have developed a set of shared socioeconomic pathways (SSPs) to complement representative concentration pathways (RCPs) for use within a scenario framework. These SSPs describe a range of plausible alternative socioeconomic developments over the 21st century at the world region level, including demographic, political, social, cultural, institutional, life‐style, economic, and technological factors (Kriegler et al., 2012; O'Neill et al., 2014, 2017).

Impact of global change including climate change can be considered an especially troubling issue for transboundary water sharing resources (Draper & Kundell, 2007). Downstream parts of the transboundary basins are often dependent upon water that flows in from outside their country boundaries, affected by changes in upstream runoff due to changing climate as well as upstream water use. This can create hydrological, social, and economic interdependencies between countries (Hoekstra & Mekonnen, 2012). It is increasingly well understood that water availability of the downstream countries is often highly dependent on upstream precipitation patterns and upstream water use (Al‐Faraj & Scholz, 2015; Drieschova et al., 2008; Veldkamp et al., 2017). Further, upstream water consumption has direct impacts on downstream water stress levels (Degefu et al., 2018, 2019; Munia et al., 2016).

While spatial dimensions are important in water scarcity assessments, so is the temporal scale. Water availability and its use, the components of water stress, can be roughly assessed in two timeframes: annual or sub‐annual/seasonal (often monthly). Both these scales are important when aiming to understand the water stress, its drivers, and potential mitigation and adaptation measures. Seasonal lack of water can often be adapted to by storing water over dry seasons with reservoirs and other types of rainwater harvesting or using groundwater resources during dry season, while aquifers are filled during rainy season. On the other hand, annual water stress requires other kind of adaptation measures, as few reservoirs, for example, are so large that they could store water over dry years. Such adaptation measures include more efficient irrigation systems (Jägermeyr et al., 2017), less resource intensive agriculture systems (Foley et al., 2011) importing food from other areas (Porkka et al., 2017) or systemic change (Varis, 2014). Therefore, both timescales are needed to understand the challenges ahead. Existing literature on climate change impacts on streamflow has emphasized change in timing rather than change in annual volumes (Clow, 2010; Stewart et al., 2005). Here we concentrate on the annual timescale while analyses on how potential future changes on seasonal water availability and water use would impact transboundary water stress are intentionally left for future studies. This also plays to the strengths of global water models, which do not yet have detailed representation of water storage and distribution systems, but can provide conservative estimates of water stress by aggregating water availability over time and space (Kummu et al., 2016).

Water scarcity can be measured in different ways (Liu et al., 2017). Physical water scarcity assesses volumes of water present within a particular region and time. Water stress specifically measures demand‐driven scarcity, that is, impacts that occur because water use is high relative to water availability (Rockström et al., 2009). It does not capture whether the population is large enough to cause water shortage (Falkenmark et al., 1989; Falkenmark et al., 2009) or whether there would be enough water, but it is not accessible due to economic water scarcity (Seckler, 1998). Also, social water scarcity is important to understand when assessing whether water is accessible to everyone, although there would be enough of it (Sullivan, 2002). The focus here is on water stress (calculated as a water use‐to‐availability ratio) because it is both a very widely used indicator (Liu et al., 2017) and it captures well a simple intuition about how water use and availability relate: When water is abundant, water use generally has less impact than when water is in short supply. Evaluation of long‐term water stress is therefore a fundamental first step in evaluating future water scarcity in transboundary basins.

The recent literature has greatly progressed in understanding the physical water scarcity in transboundary basins. For example, Munia et al. (2016) identified that about 0.95–1.44 billion inhabitants in transboundary basins are under stress because of local water consumption, while upstream water use increased the stress level considerably in many areas, affecting approximately 0.29–1.13 billion people. Degefu et al. (2019) estimated that about 2.12 billion people experience changes in water stress level for at least 1 month of the year as the result of upstream water use. Munia et al. (2018) further examined transboundary water dependency based on the concept that a basin area is dependent on upstream inflows if it requires those inflows to avoid water scarcity (e.g., stress and shortage) and its associated impacts under present water use and water availability conditions.

Little is known, however, about how the situation has developed over the past decades, and how future development would impact on the transboundary water stress levels as well as dependency dynamics. While increase in water demand is often cited as a key factor affecting water scarcity in most transboundary river basins (Degefu et al., 2016), concerns about water availability are also considered to be one of the most important issues for international cooperation and conflict concerning shared water basins (Beck et al., 2014). In the case of transboundary basins, a basin's water availability is composed of local availability and upstream inflows, which are reduced by upstream water consumption. Changes in climate would impact both local and upstream water availability. As a result, transboundary water scarcity is not only limited to the local demand and local availability but to upstream water consumption and upstream inflows, which, together, can be considered the proximate drivers of water stress. The role of these drivers in past development of water stress in transboundary basins, or in future development scenarios, is not yet assessed in literature and thus presents a considerable research gap regarding which of the drivers should be prioritized in future water management planning.

In this analysis, we thus aim to assess how water stress has developed in global transboundary basins in the past and how it may change in future scenarios. The analysis specifically identifies the drivers of water stress indicators in a transboundary contexts and thus what role each of these drivers would play in overall change. The results provide new information and knowledge on drivers of water scarcity in global transboundary basins, both for the past and the future, and hence potential priorities for adaptation.

## 2 Materials and Methods

We first assessed how the water stress and its drivers have changed over the past decades, between the 1980s and 2010s, and then examined the potential changes up to the 2050s. To estimate the changes over time in water availability and consumption, we used the available output data from global hydrological models. For the future, we created scenarios using projected climate change scenarios (RCPs) (Van Vuuren et al., 2011) and shared socioeconomic pathways (SSPs) (O'Neill et al., 2014).

As noted in section 1, we study water scarcity measured by water stress, using the use‐to‐availability ratio (Rockström et al., 2009), which focuses on demand‐driven physical water scarcity. Higher water stress means that a higher proportion of available water is being used, which means that it becomes more difficult to access water (e.g., requiring infrastructure) and that it becomes increasingly difficult to meet all water consumers' needs; there is less water for the environment, more potential for conflict, and a greater need for cooperation (Kummu et al., 2016). Therefore, it is considered that the higher the water stress, the more vulnerable is the population to decreases in water availability (Van Beek et al., 2011; Wada et al., 2011).

We investigated changes in water stress based on a two‐part analysis. In the first step, we performed a basic analysis comparing water use and availability, and resulting water stress, in the past, present, and future. We quantified the contributions of water use and availability to stress combining results from multiple models, using the ensemble median, and then investigate the uncertainty in these three variables. The results highlight which drivers dominate changes in water stress. In the second part of the analysis, we assessed how local availability, upstream inflows, and upstream consumption contribute to net water availability in a subbasin. This distinction allowed us to examine how relationships between transboundary subbasins have changed in the past and might change in future. Finally, we draw on Munia et al. (2018) to identify subbasins that are dependent on upstream inflows in these future scenarios and which therefore require increased attention to the management of transboundary relations.

## 3 Data

We applied four scenarios developed under the water futures and solutions (WFaS) initiative (Wada et al., 2016) and consisting of combinations of RCP and SSP scenarios, as shown in Table 1.

**Table 1:** Scenarios Used in This Analysis, Constructed as Combinations of Shared Socioeconomic Pathways (SSPs) and Representative Concentration Pathway

SSPs	RCPs	Scenarios
*SSP1: sustainability*—*taking the green road*	*RCP 2.6*	*SSP1‐RCP2.6* (*Scenario 1*, *S1*)
Rapid technology, high environmental awareness, and low energy demand. Medium‐high economic growth with low population	Peak in radiative forcing at ∼3 W m^−2^ (∼ 490 ppm CO_2_ eq) before 2,100 and then decline (the selected pathway declines to 2.6 W m^−2^ by 2,100)	
	*RCP 4.5*	*SSP1‐RCP4.5* (*S2*)
	Stabilizes radiative forcing at 4.5 W m^−2^ in the year 2,100 without ever exceeding that value	
*SSP2*: *middle of the road*	*RCP 6.0*	*SSP2‐RCP6.0* (*S3*)
Most economies are politically stable. Markets are globally connected, but they function imperfectly. Slow progress in achieving development goals of education, safe water, and health care.	Stabilization without overshoot pathway to 6 W m^−2^ (∼⃒850 ppm CO_2_ eq) at stabilization after 2,100	
*SSP3*: *regional rivalry*—*a rocky road*		*SSP3‐RCP6.0* (*S4*)
Slow technology development. Reduced trade, very slow economic growth, and very high population		

*Note*. Scenario SSP1‐RCP2.6 was selected based on the newly adopted Paris Agreement under the United Nations Framework Convention on Climate Change (Paris Agreements, 2015), and scenarios SSP1‐RCP4.5, SSP2‐RCP6.0, and SSP3‐RCP6.0 were selected based on the water futures and solutions (WFaS) initiative (Wada et al., 2016).

For each scenario, we used an ensemble of 20 model runs combining four global hydrological models (GHMs) and five global climate models (GCMs) (see Table 2) to obtain decadal water availability and water consumption data from 1971 to 2050. Due to the varying model output availability for historical and future periods, we needed to collect data from a few different sources—namely, inter‐sectoral impact model intercomparison project (ISIMIP) fast‐track for water availability and irrigation, and ISIMIP 2a and WFaS for other water consumption—as detailed below and in Table 2. ISIMIP provides a comprehensive collection of state‐of‐the‐art global hydrological models designed to capture both water availability and human water consumption at a 0.5 degree grid resolution (Veldkamp et al., 2017). All the models are carefully calibrated and validated against time series from several thousand discharge stations, available at Global Runoff Data Centre (www.bafg.de/GRDC), across the globe (Mueller Schmied et al., 2016; Veldkamp et al., 2018; Wartenburger et al., 2018; Zaherpour et al., 2018). In this analysis, we used simulations from H08, LPJmL, PCR‐GLOBWB, and WaterGAP, as these are the only four GHMs that provide irrigation water consumption estimates at global scale. The GCMs provided for these GHMs were GFDL‐ESM 2M, HadGEM2‐ES, IPSL‐CM5A‐LR, MIROC‐ESM‐CHEM, and NorESM1‐M.

**Table 2:** Summary of Data Used

Impact models/GHMs	Historical 1980 (average of 1971–1980), present 2010 (average of 2001–2010)				Future 2050 (average of 2041–2050)				Global circulation models (GCMs)	Scenarios
	Irrigation	Domestic	Industrial	Availability	Irrigation	Domestic	Industrial	Availability		
WaterGAP	ISIMIP Fast Track	Provided by University of Kassel	Provided by University of Kassel	ISIMIP Fast Track	ISIMIP Fast Track	WFaS	WFaS	ISIMIP Fast Track	GFDL‐ESM 2M HadGEM2‐ES IPSL‐CM5A‐LR MIROC‐ESM‐CHEM NorESM1‐M	SSP1‐RCP2.6 SSP1‐RCP4.5 SSP2‐RCP6.0 SSP3‐RCP6.0
LPJmL	ISIMIP Fast Track	NA^a^	NA^a^	ISIMIP Fast Track	ISIMIP Fast Track	NA^a^	NA^a^	ISIMIP Fast Track		
H08	ISIMIP Fast Track	ISIMIP2a	ISIMIP2a	ISIMIP Fast Track	ISIMIP Fast Track	WFaS	WFaS	ISIMIP Fast Track		
PCR‐GLOBWB	ISIMIP Fast Track	ISIMIP2a	ISIMIP2a obtained from IIASA	ISIMIP Fast Track	ISIMIP Fast Track	WFaS	WFaS	ISIMIP Fast Track		

^a^ For LPJML, the average of other estimates is used for domestic and industrial water consumption.

The GCMS were used for both past and future water availability to ensure consistency of climate conditions. Water availability estimates from climate reanalysis data (e.g., from ISIMIP2a) would provide a more accurate historical estimate but are not directly comparable with GCM results. In this case, consistency within each scenario to capture changes over time was more important than fidelity to historical conditions, so the GCM results were directly adopted rather than attempting some correction, for example, using delta change methods. We used the monthly runoff (*mrro*) parameter under natural conditions for water availability, as our analysis method then separately takes into account upstream water consumption. ISIMIP Fast Track data were used primarily based on availability of GCM data, at the time project started, for the required variables for all RCPs for the period 1980–2050.

For estimation of water use, we used water consumption data. Water consumption is the water use that permanently removes water from the immediate water environment by abstracting, evaporating, or consuming it. The calculation may thus understate the impact of water use as consumptive use assumes full availability of potential return flows from water withdrawals. Water consumption estimates were used for three key components: irrigation, industrial, and domestic water consumption. Livestock water consumption was not taken into account in this study. Historical irrigation water consumption estimates were obtained directly from the ISIMIP Fast Track data product, separately for each of the GHM data sets. For historical domestic and industrial water consumption, we used the data from the ISIMIP2a historical validation experiment for H08 and PCR‐GLOBWB. For WaterGAP, historical domestic and industrial potential water consumption estimates were provided by University of Kassel, both compatible with the ISIMIP2a setup. The ISIMIP historical validation experiment was expected to provide the most reliable estimate of historical domestic and industrial water consumption. On the other hand, irrigation water consumption is highly dependent on climate and is therefore needed to be obtained from the Fast Track product using the GCMs, rather than from ISIMIP2a using historical climate observations. LPJmL does not provide domestic and industrial water use estimates, so we used the average of the three other models. LPJmL is included because it provides an independent estimate of irrigation water use under climate change, which is by far the largest water consumption sector (Kummu et al., 2016).

For future irrigation water consumption estimates, we also used the ISIMIP Fast Track data. It should be noted that this data set does not capture the potential to improve agricultural water use efficiency or the potential expansion of the irrigated area (Wada et al., 2013). These two drivers partially cancel each other and are influenced by complex drivers (Kummu et al., 2017), such that developing and running models for future irrigation scenarios is a major endeavor—currently being tackled by the WFaS initiative. For example, a previous effort by Hanasaki et al. (2013) scales up irrigated area within grid cells without considering land suitability or new irrigation projects, but with estimates of altered crop intensity and irrigation efficiency. As it stands, we considered that current irrigation extent and technology provides a fit for purpose estimate of future irrigation water use accounting for the combined impact of future irrigation extent and technology. Future water consumption for domestic and industrial sectors was taken from the water futures and solutions (WFaS) initiative (Wada et al., 2016) at the International Institute for Applied Systems Analysis (IIASA) for WaterGAP, H08, and PCR‐GLOBWB. For LPJML, the average of other estimates is again used, given that the model does not provide these data (see Table 2).

Transboundary basins were identified on a 30 arc‐min grid in the form of subbasin areas (SBAs). SBAs were defined by breaking up the drainage direction grid where it flows across country (and shared zone) boundaries, effectively yielding a mesh of river basin and country boundaries. Upstream‐downstream relationships between these SBAs were defined by the flow direction data set. A more detailed description of the construction of the basin‐country raster can be found in Munia et al. (2018).

**Figure 1:**
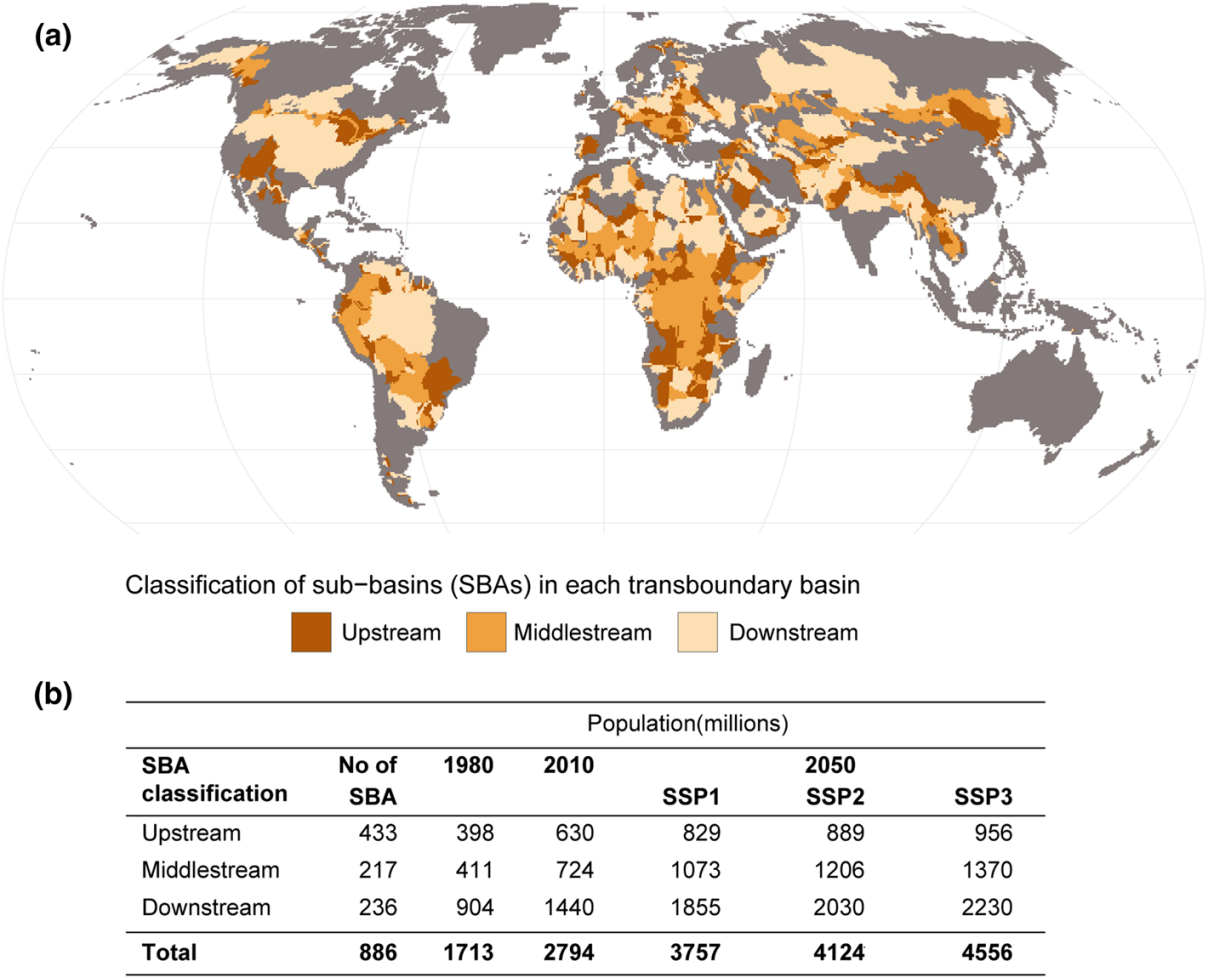
Classification of subbasin areas (SBAs). Identified upstream, middle stream, and downstream SBAs (a) and population within each transboundary basin and future shared socioeconomic pathway (SSP) scenario (b). The SBA delineation is adopted from Munia et al. (2018).

To estimate the population impacted by water stress, we used gridded population data from the HYDE data set, providing population from 1980 to 2050 for each SSP scenario (Klein Goldewijk et al., 2010). The data were first aggregated from 5 to 30 arc‐min resolution and then for each SBA for every year over the 50‐year study period (Figure 1).

### 3.1 Methods, Part I: Water Stress Assessment

#### 3.1.1 Calculation of Change in Water Stress

Local water consumption under each scenario was calculated separately by summing up the three water consumption sectors (industrial, domestic, and irrigation) and then taking the decadal ensemble median across all GHM and GCM combinations (Table 2). Local water availability was also calculated taking the decadal ensemble median of local availability across all combinations of GHMs and GCMs. We then aggregated both local consumption and availability to SBA scale. Water availability for each SBA was obtained by summing local water availability and upstream inflows, and subtracting upstream water consumption from this. Then we calculated the water stress as the ratio of local consumption (*LocalWC*) to availability (*AA*) of each subbasin (see, e.g., Falkenmark et al., 2007; Kummu et al., 2016):

(1) $$\text{stress}\;=\;\frac{{\text{LocalWC}}_{\text{year}}}{{\mathrm{AA}}_{\text{year}}}.$$

Changes in stress from one time period to another were calculated simply by subtracting the stress level of one time period from another. In this analysis, we calculated the change in stress from 2010 to 2050, and for comparison, we looked at the past change from 1980 to 2010. Note that because decadal medians of use and availability were used, the labels 1980, 2010, and 2050 correspond to the periods 1971–1980, 2001–2010, and 2041–2050, respectively.

The equation for change in stress then calculates the difference between consecutive time steps:

(2) $$\Delta \text{Stress}\;=\;{\text{Stress}}_{t+1}-{\text{Stress}}_{t}.$$

#### 3.1.2 Relative Contribution of Consumption and Availability to Change in Stress

The immediate drivers of water stress for every SBA are *LocalWC* and *AA*, according to Equation 1. We calculated the contribution of these two drivers in past and future changes in stress using the equations below. In each equation, a single variable was varied, keeping the other constant at the level of the first time step (*t*). Contributions were then calculated proportional to the total change in stress, varying both variables at once:

Equation for the contribution of *LocalWC*:

(3) $$\Delta {\text{Stress}}_{\text{LocalWC}}\;=\;\left(\frac{{\text{LocalWC}}_{t+1}}{{\mathrm{AA}}_{t}}-\frac{{\text{LocalWC}}_{t}}{{\mathrm{AA}}_{t}}\right)/\left|\left(\Delta \text{Stress}\right)\right|,$$

Equation for contribution of *AA*:

(4) $$\Delta {\text{Stress}}_{\text{TotalAA}}\;=\;\left(\frac{{\text{LocalWC}}_{t}}{{\mathrm{AA}}_{t+1}}-\frac{{\text{LocalWC}}_{t}}{{\mathrm{AA}}_{t}}\right)/\left|\left(\Delta \text{Stress}\right)\right|.$$

To more clearly highlight the relative importance of drivers, we then normalized the effect of these water stress drivers in each SBA, by dividing each driver by the maximum contribution in an SBA in question, such that the driver with the greatest effect is assigned a value of 1:

(5) $$\text{Relative Contribution of each driver}\;=\;\frac{\text{contribution of each driver}}{\text{Maximum}\left(\left|\left(\Delta {\text{Stress}}_{\mathrm{AA}},\Delta {\text{Stress}}_{\text{LocalWC}}\right)\right|\right)}.$$

#### 3.1.3 Uncertainty Estimates

To assess the uncertainty across GHMs and GCMs, we calculated the ratio of median absolute deviation (MAD) to the median for the three variables in each SBA: *LocalWC*, *AA*, and *Stress*. This is analogous to the coefficient of variation—It provides a relative measure of spread of results. Since we applied four different GHMs and five different GCMs, we calculated the MAD from 20 values, separately for each of the three variables and for each SBA. MAD around the median of the modeled values was chosen to represent the dispersion of values because it is generally recognized as a more robust estimator of variability than standard deviation due to being less sensitive to outliers (Hoaglin et al., 1983). Normalizing the resulting MAD value by dividing it with the median makes it easier to compare subbasins of different sizes since the result is a relative number. High MAD/median ratios indicate high dispersion of modeled values for a particular variable in an SBA; thus, there is less certainty about the assessed variable. Low MAD/median ratio, in turn, implies that the distinct GCM/GHM combinations provide more equal values for a particular variable, which means that there is more certainty about the assessed variable in that SBA.

To understand whether a single model is responsible for extreme estimates, we analyzed the domination of GHMs and GCMs, aiming to understand the origin of highly deviating estimates. The results of this analysis are provided in the Supporting Information but briefly discussed in section 4. We considered that the results of an SBA were dominated by an individual GHM or GCM if one GHM or GCM gives the largest or smallest estimates for a variable in every time step.

### 3.2 Methods, Part II: Role of Local and Upstream Changes

Returning to the equation of water stress (Equation 1), and its drivers, *LocalWC* and *AA*, we note that while local consumption is only impacted by an SBA's local water use, availability in a transboundary SBA comprises the following components:

1. Local availability (*LocalAA*)
2. Upstream inflows (*UpAA*)
3. Reduction due to upstream water consumption (*UpWC*)

In this part of the analysis, we assessed how these different components of availability contribute to past and future changes in SBAs water availability. We also examined the different categories of dependency to understand whether subbasins will become more dependent on upstream inflows in future, using the dependency framework developed by Munia et al. (2018).

#### 3.2.1 Relative Changes in Components of Water Availability

Given that in a transboundary setting upstream actions only affect downstream stress through downstream availability, it is useful to compare the size of each component of availability. For comparison, we also looked at change in local consumption. We therefore calculated the change in each component:

(6) $$\Delta \text{LocalAA}\;=\;{\text{LocalAA}}_{t+1}-{\text{LocalAA}}_{t},$$

(7) $$\Delta \text{UpAA}\;=\;{\text{UpAA}}_{t+1}-{\text{UpAA}}_{t},$$

(8) $$\Delta \text{LocalWC}\;=\;{\text{LocalWC}}_{t+1}-{\text{LocalWC}}_{t},$$

(9) $$\Delta \text{UpWC}\;=\;{\text{UpWC}}_{t+1}-{\text{UpWC}}_{t}.$$

We then compared the relative magnitude of changes of these drivers with each other separately within each SBA, such that the driver with the highest change relative to others is assigned a value of 1:

(10) $$\text{Relative changes of each driver}\;=\;\frac{\Delta \text{Driver}}{\text{Maximum}\left(\left|\left(\Delta \text{LocalAA},\Delta \text{UpAA},\Delta \text{LocalWC},\Delta \text{UpWC}\right)\right|\right)}.$$

#### 3.2.2 Change in Upstream Dependency

**Figure 2:**
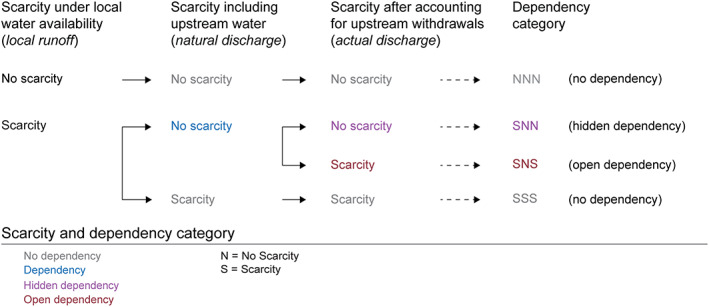
Definition of upstream water dependency categories. Dependency categories are obtained by summarizing three letter codes representing the scarcity category using local availability, natural discharge, and actual discharge respectively (modified from Munia et al., 2018).

The analytical framework developed in Munia et al. (2018) was used, which draws on ideas of regime shifts from resilience literature, to understand the transition between cases where water scarcity is or is not experienced, depending on whether sufficient water from upstream is or is not available. Dependency means that water from upstream is needed to avoid scarcity. Based on the role of upstream inflows and withdrawals, a region might experience (i) no dependency if stress is not affected by upstream inflows, (ii) “hidden” dependency if stress is altered by upstream inflows but not by upstream water withdrawal, or (iii) “open” dependency if stress is altered after accounting for upstream water withdrawals. The typology developed in Munia et al. (2018) is presented in Figure 2.

The transitions and typology provided in the analysis give a basic level of guidance for mitigation and prevention of scarcity in a region. In this analysis, we used this framework to calculate the number and population of SBAs under different dependency categories for future scenarios in order to differentiate the role of local and upstream changes in future water stress.

To assess the dependency, we calculated three types of water availability for each of the SBA, corresponding to

$$\begin{array}{l} \text{Local}\;\text{runoff}\;=\;{\text{localAA}}_{t},\\ \text{Natural}\;\text{dichagre}\;=\;{\text{localAA}}_{t}+{\text{UpAA}}_{t}\\ \text{Actual}\;\text{disch}\arg e\;=\;{\text{localAA}}_{t}+{\text{UpAA}}_{t}-{\text{UpWC}}_{t}. \end{array}$$

We then calculated the upstream dependency condition in terms of water scarcity (here water stress) for each scenario. Water stress was deemed to occur when the use‐to‐availability ratio exceeded 0.2 (Falkenmark et al., 2007).

## 4 Results

Results are split into two parts, as described in the methods section. In the first part, we focus on changes in stress between past (1980s), present (2010s), and future conditions (2050s), the drivers of these changes and uncertainty involved. In the second part, we specifically focus on the role of local versus upstream changes, given this is a fundamental issue in transboundary basins. The results also differentiate effects of availability versus use, which are most closely associated with the global issue of climate change and the predominantly local issue of socioeconomic development, respectively. In the main text, we include figures for one scenario, SSP3‐RCP6.0, as the main findings do not largely differ between scenarios. The results for other scenarios are presented in the Supporting Information. However, tabulated results are shown for all scenarios in the main text.

### 4.1 Part I: Water Stress Assessment

#### 4.1.1 Changes in Stress

**Figure 3:**
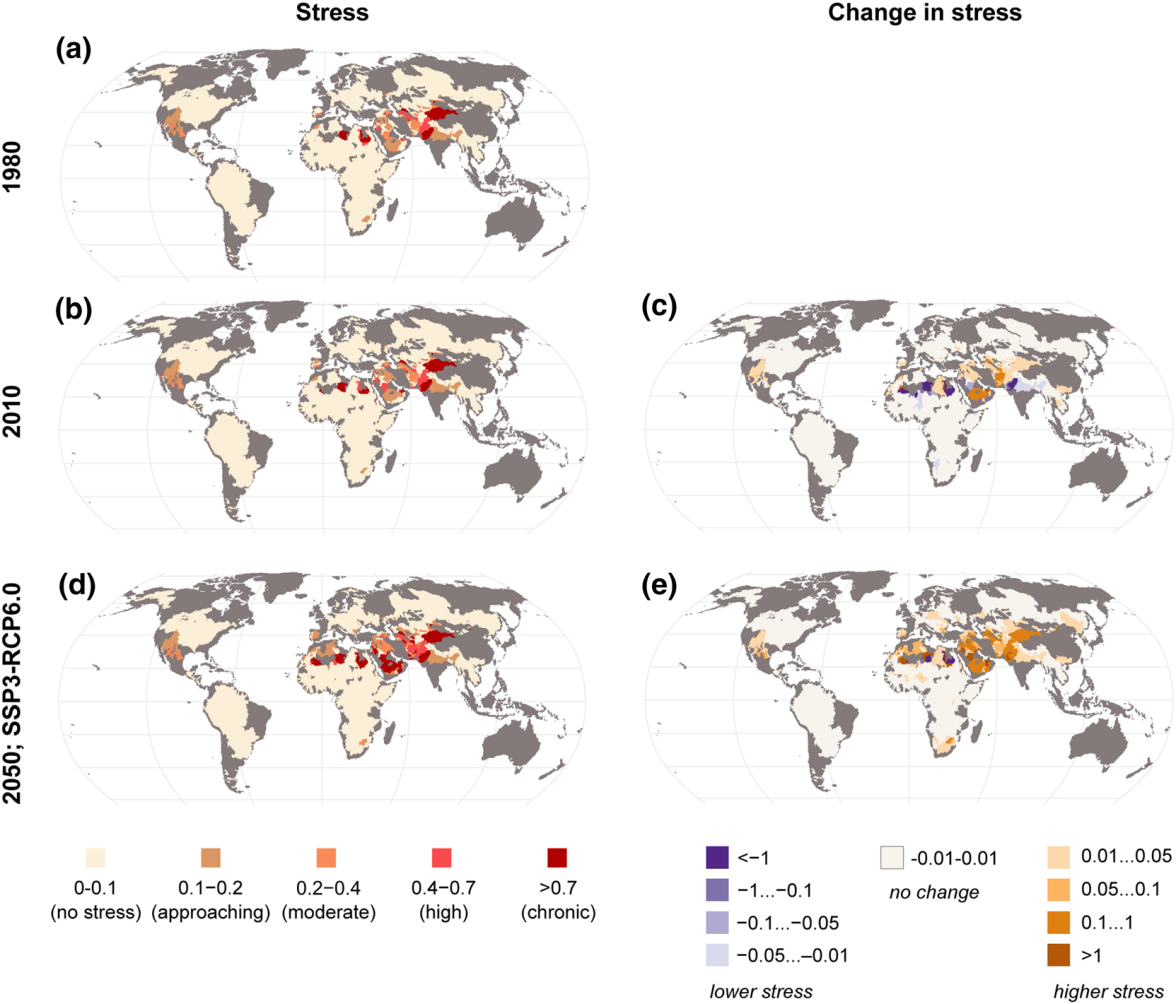
Water stress in transboundary subbasin areas (SBAs) for past (1980s) (a), present (2010s) (b), and future conditions (2050) (d). Absolute changes in stress level from 1980 to 2010 (c) and from 2010 to 2050 (e) under future scenario SSP3‐RCP6.0. Negative change values mean that stress level has decreased and positive ones means stress level has increased. Given stress is measured using a use‐to‐availability ratio, changes in stress can be interpreted as proportions of availability. For the future, one of the SSP‐RCP scenarios is shown here (see supporting information Figure S1 for other scenarios).

Change in stress was calculated for 246 international transboundary basins which were divided into 886 SBAs based on country borders (as well as shared zones along those borders) (Figure 3). Our findings show that SBAs in Asia, Middle East, and North Africa regions were the main areas identified as having moderate to extreme stress under past (1980) and present (2010) conditions. Stress is projected to intensify in areas already under stress in all future scenarios (Figure 3 and supporting information S1).

Changes in stress level (both increase and decrease) from one time period to another are observed mostly in Central and Southeast Asia and northern Africa for past years (1980 to 2010) and also for all the future (2050) scenarios (Figures 3c and 3e and supporting information Figure S1). In the case of SSP1‐RCP2.6, some SBAs are expected to experience decreases in stress level, for example, in Asia (Indus) and in northern Africa (Nile), while under SSP3‐RCP6.0, decreases in stress level are observed only in a few SBAs in northern Africa. In these cases, decrease in stress level is because of decreases in water consumption (e.g., population stabilization and efficiency gains) and increase in water availability (e.g., wetter climate).

When assessing the impacted population, we found that population under water stress (Stress >0.2) more than doubled (185 million people) from 1980 to 2010 (Table 3). For future (2050) scenarios, total population living under water stress is expected to increase from present conditions (2010) by almost 50% (+175 million) under SSP1‐RCP2.6 and more than 100% (+380 million) under SSP3‐RCP6.0 scenario. The number of SBAs approaching stress decreases as stress becomes more widespread, though the population in those SBAs still increases, consistent with population growth in most SBAs.

**Table 3:** Number and Population of Subbasins Under Stress for the Past (1980), Present (2010), and Future (2050

		Number of SBA		Population (millions)	
Year		Approaching stress (0.1–0.2)	Stress (>0.2)	Approaching stress (0.1–0.2)	Stress (>0.2)
Past 1980		78	74	263	150
Present 2010		92 (+14)	84 (+10)	393 (+130)	335 (+185)
Future 2050	SSP1‐RCP2.6	87 (−5)	93 (+9)	490 (+97)	510 (+175)
	SSP1‐RCP4.5	82 (−10)	95 (+12)	420 (+27)	519 (+185)
	SSP2‐RCP6.0	83 (−11)	100 (+17)	512 (+119)	612 (+278)
	SSP3‐RCP6.0	88 (−4)	101 (+18)	540 (+147)	714 (+380)

*Note*. In brackets, we present the changes from the immediately preceding time step.

#### 4.1.2 Contribution of Stress Drivers to Change in Stress

**Figure 4:**
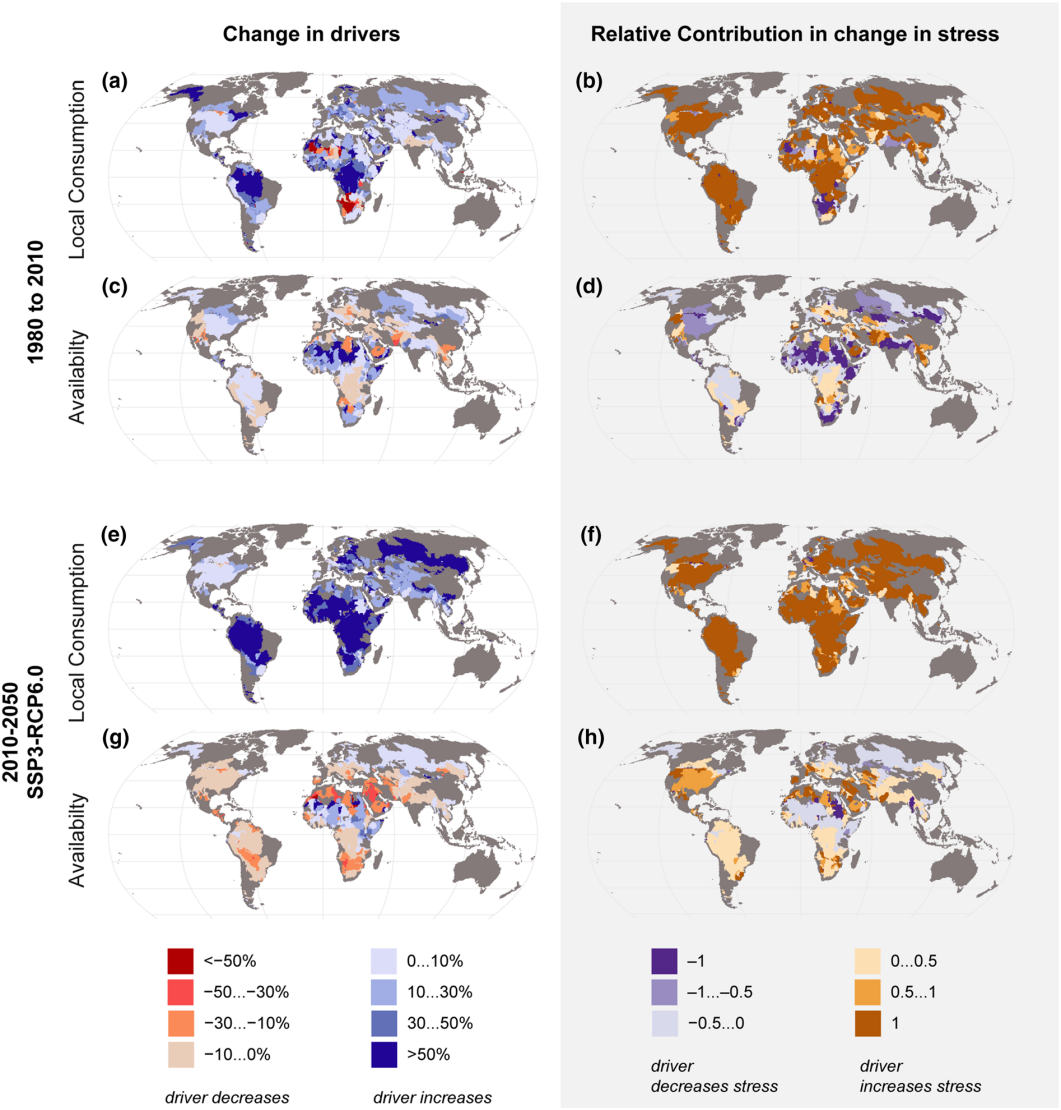
Drivers of water stress. Changes in availability and local consumption in 2010 compared to 1980 (a, c) and in 2050 compared to 2010 under one scenario (e, g). The result is presented as percentage (%) changes. Relative contribution of local water consumption (*LocalWC*) versus water availability (*AA*) to change in stress in 2010 compared to 1980 (b, d) and 2050 compared to 2010 (f, h). The result is presented in the scale of −1 to +1, where −1 refers to maximum contribution in decreasing stress and +1 refers to maximum contribution in increasing stress (see methods section). For the future, one of the SSP‐RCP scenarios is shown here (see supporting information Figure S2 for other scenarios).

To assess which of the drivers would have the most influence on change in water stress, we compared the changes in stress drivers (*LocalWC*, *AA*) between past and present conditions (1980 and 2010) and between present conditions and future scenarios (2010 and 2050). Local water consumption (*LocalWC*) is estimated to increase rather than decrease almost everywhere in both present and future (Figure 4 and supporting information Figure S2) scenarios. When comparing the changes from 1980 to 2010, only a few SBAs in Southeast Asia and Africa can be identified where *LocalWC* has decreased. For future scenarios, SBAs within river basins like Indus and St Lawrence under SSP1‐RCP2.6 and Rhine under both SSP1‐RCP2.6 and SSP3‐RCP6.0 are identified where *LocalWC* is expected to decrease.

In the case of water availability (*AA*), our results identified decreases in many SBAs in all scenarios (Figure 4a and supporting information Figure S2a). For some SBAs, results for changes in *AA* vary in different scenarios—for example, in Ganges‐Brahmaputra, *AA* is predicted to increase under the SSP1‐RCP2.6 scenario and decrease under the SSP3‐RCP4.5 scenario. For Europe and South America, the direction of change is somewhat consistent for all the future scenarios.

When looking at the contribution of *AA* and *LocalWC* in changes of stress level (Figure 4 and supporting information Figure S2; see also supporting information Figure S3), we found that the principal cause of increasing water stress is local consumption while decreasing water stress (where it occurs) is mainly related to increasing water availability due to climate change for both past and future scenarios. In most SBAs, the contribution of changes in availability to change in stress is mostly minor. However, some SBAs, such as Colorado and Tigris under SSP1 + RCP2.6 scenario, Indus and Aral Sea under SSP3 + RCP 6.0 scenario, and some parts of Europe under both aforementioned scenarios are identified as basins where availability has the largest contribution to changes in stress level (Figure 4 and supporting information Figure S2).

**Figure 5:**
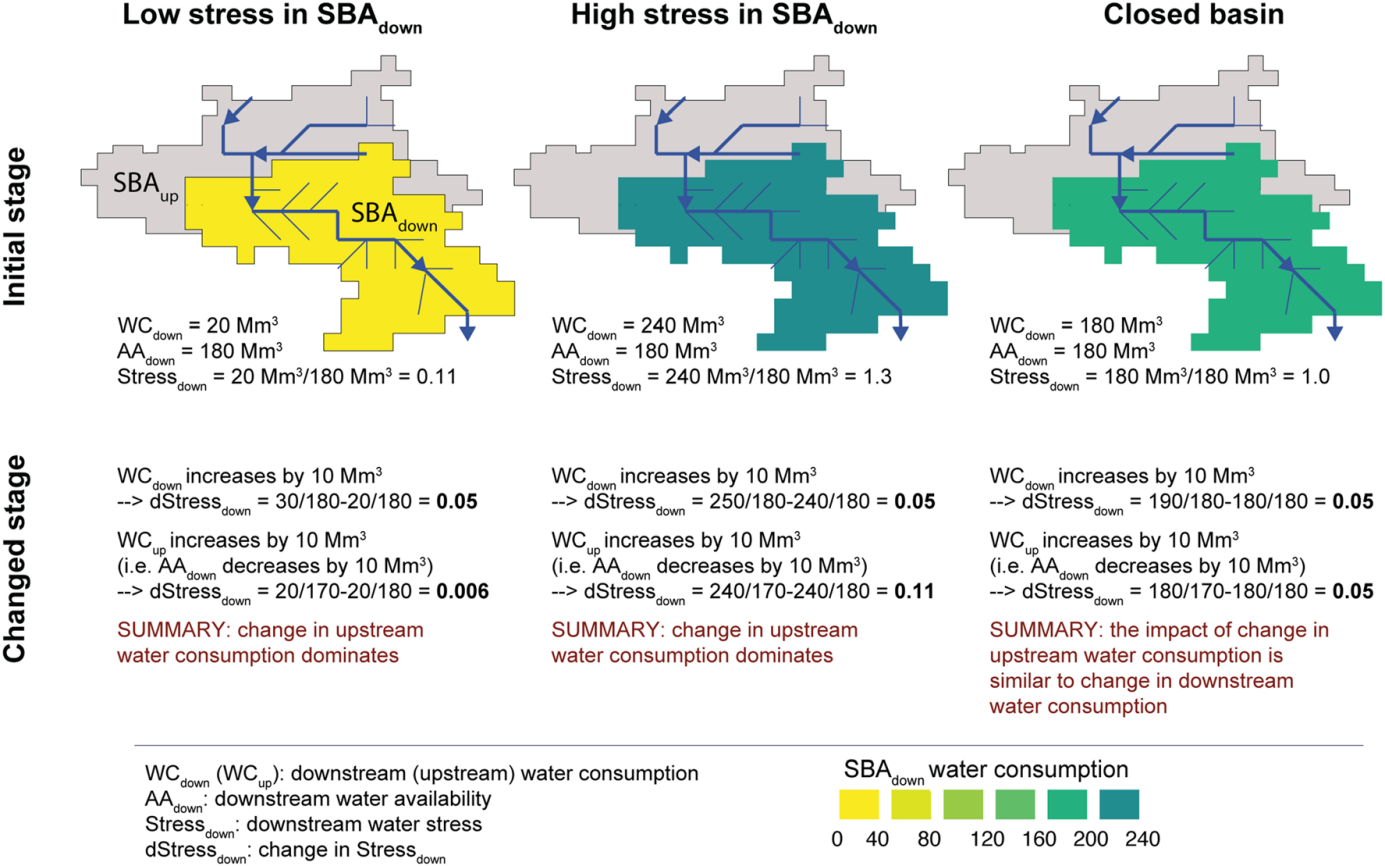
Example basin, illustrating the impact of local water consumption increase versus upstream consumption increase. The impact is shown to vary depending on the stress level; here three cases are shown. WC refers to water consumption, AA to water availability, and Stress to water stress.

It should be noted that depending on the stress level, the water stress indicator gives a different weight to consumption and availability (see Equation 1and Figure 5). An example shown in Figure 5 illustrates that in basins with low stress, the increase in local water consumption results in much higher change in stress than a similar increase in upstream water consumption. However, in basins with high stress (Stress >1), the case is the opposite, that is, an increase in upstream consumption dominates the change. In closed basins (downstream subbasin area uses all its water availability) the impact of increase in local and upstream water consumption is similar.

As in most of the basins the water stress is rather low (Figure 3a), the changes in local consumption dominate the change in stress levels over the availability (on which upstream water consumption or changes in either downstream or upstream runoff impact). The weak effect of availability on stress is further compounded by our findings that consumption is likely to change more than availability over time (Figure 4). The former may be doubled or more in developing regions, whereas change in annual runoff seldom exceeds 40% anywhere in the world due to climate change (Figure 4).

#### 4.1.3 Uncertainty and Extreme Predictions for Water Consumption and Availability

**Figure 6:**
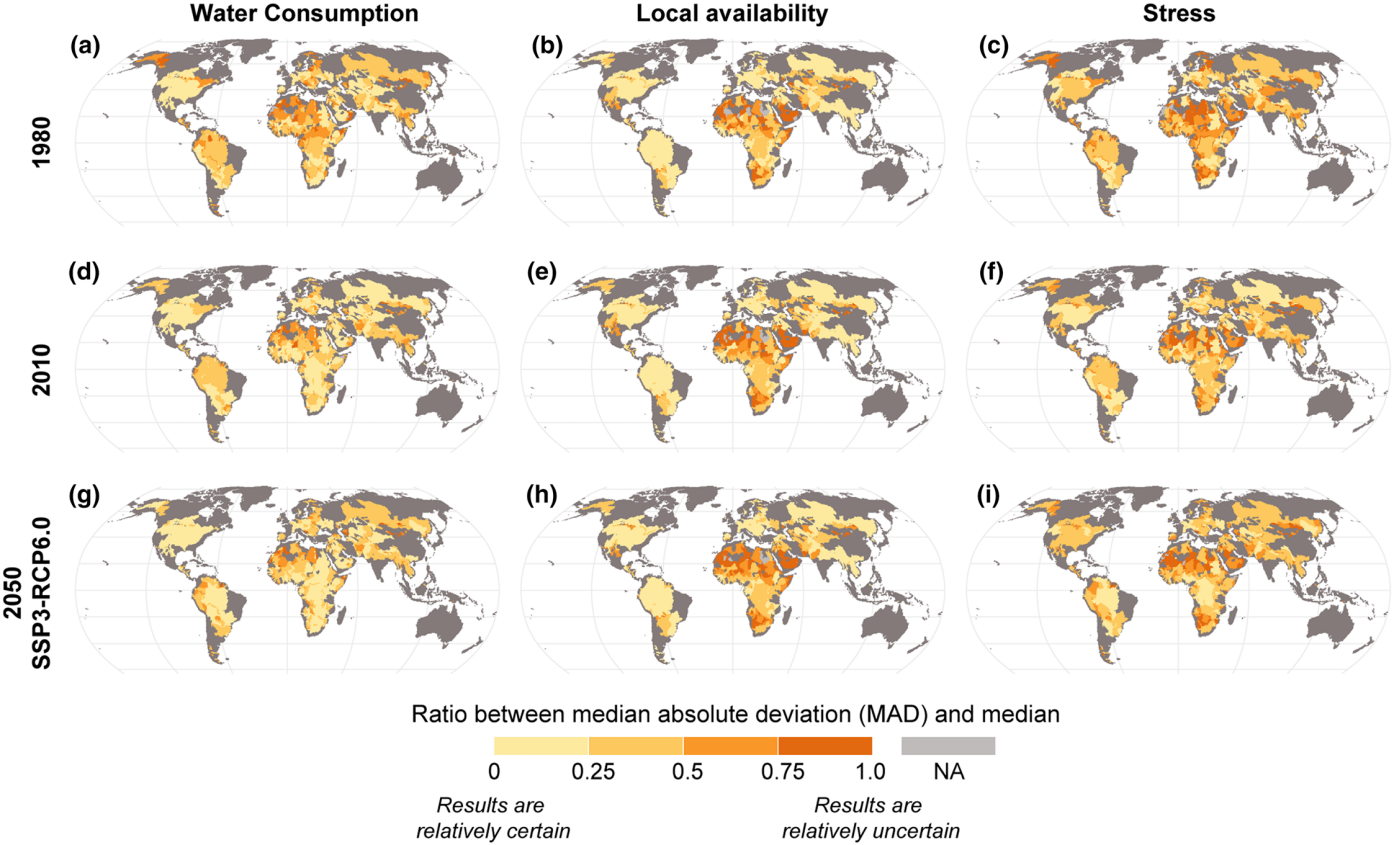
MAD/median ratio for local water consumption (a, d, j), water availability (b, e, k), and water stress (c, f, l). If the value is zero, it means that at least 50% of estimates are identical to the median and there is little uncertainty between estimates. If the value is 1, it means that the estimates have both very small and large values, and their deviation from the median is large. NA indicates where the median itself is zero. For the future, one of the SSP‐RCP scenarios is shown here, see supporting information Figure S4 for other scenarios).

The preceding results are based on an ensemble median of *LocalWC*, *AA*, and *Stress* for each subbasin. We used MAD/median ratio to explore the spread across the ensemble, thus reflecting uncertainty between our ensemble members. We found that dispersion of the availability (*AA*), consumption (*LocalWC*), and water stress values in some SBAs is relatively high—over 0.75—for past, present, and all the future scenarios (see Figure 6 and supporting information Figure S4). This implies relatively high uncertainty in all variables. Low MAD/median ratio implies that GCM‐GHM combinations agree on a particular quantity so there is more certainty about the result in this subbasin. For example, SBAs in North and South America have a dispersion value of almost zero for *LocalWC* and *AA*, indicating less uncertainty than, for example, in large parts of the MENA (the Middle East and North Africa) region where the dispersion is more than 0.50.

For stress, dispersion in the estimates arises both from dispersion in *LocalWC* and *AA* values. Dispersion is more than 0.50 in parts of Asia, Africa, Europe, and South America (Figure 6). The largest dispersion (>0.75) is found mostly in northern Africa for all the time steps and scenarios.

To assess the sensitivity of our results to the use of different ensemble of GHMs and GCMs, in additional analyses (presented in Supporting Information) we investigated the origin of the minimum and maximum values of variables. Supporting information Figures S7 and S8 show the overall domination maps of the GHMs and GCMs used in the analysis, that is, which GHMs or GCMs were consistently responsible for the maximum or minimum estimate in the ensemble for an SBA, across all time steps. For both availability and water consumption, none of the GCMs show any domination (supporting information Figure S7), while for GHMs we found domination in multiple SBAs (supporting information Figure S8). GHM domination is the most prevalent in water consumption data: for example, maximum estimates of water consumption are provided mostly by PCR‐GLOBWB across the world while minimum estimates are provided by WATERGAP, LPJML, and H08 depending on the SBA (supporting information Figure S8). In this analysis we did not look at the source of inter‐model difference between different sectors of water use. The source of inter‐model difference is distinct between different sectors of water use, which is the predominant difference among models as previously discussed in model intercomparison projects (Mueller Schmied et al., 2016; Zaherpour et al., 2018).

### 4.2 Part II: Role of Local and Upstream Changes

In the context of transboundary water management, the roles of local versus upstream changes in water use and availability are important. Upstream changes, however, only manifest themselves in the availability term of the stress equations, and as we demonstrated under section 3.1, availability has in most cases a much lower effect on stress than local water consumption. On one hand, this emphasizes the importance of managing local demand in order to avoid stress. On the other hand, even if availability is in most SBAs the less important term, it is still useful for transboundary water managers to understand how potential changes in upstream availability and upstream water consumption affect local availability. If changes in local availability are driven primarily by changes in natural runoff, this is mostly beyond the control of local decision makers—they can plan adaptation and do their share to reduce CO_2_ emissions to mitigate climate change. At the annual timescale used in this analysis (averaged over a decade), the potential for storage to maximize the runoff captured has already been taken into account. Any further management to increase capture of runoff would require quite substantial infrastructure, such as interbasin water transfers, or extensive use of nature‐based solutions such as wetlands, lakes, and small ponds. On the other hand, if changes in local availability are driven by changes in upstream water consumption, they can be influenced by negotiations. And as a benchmark, if changes in local consumption still had a greater effect than local availability even outside the stress equation, then this is a strong signal in favor of local actions to improve water use efficiency and reduce water use.

#### 4.2.1 Relative Changes in Components of Water Availability

**Figure 7:**
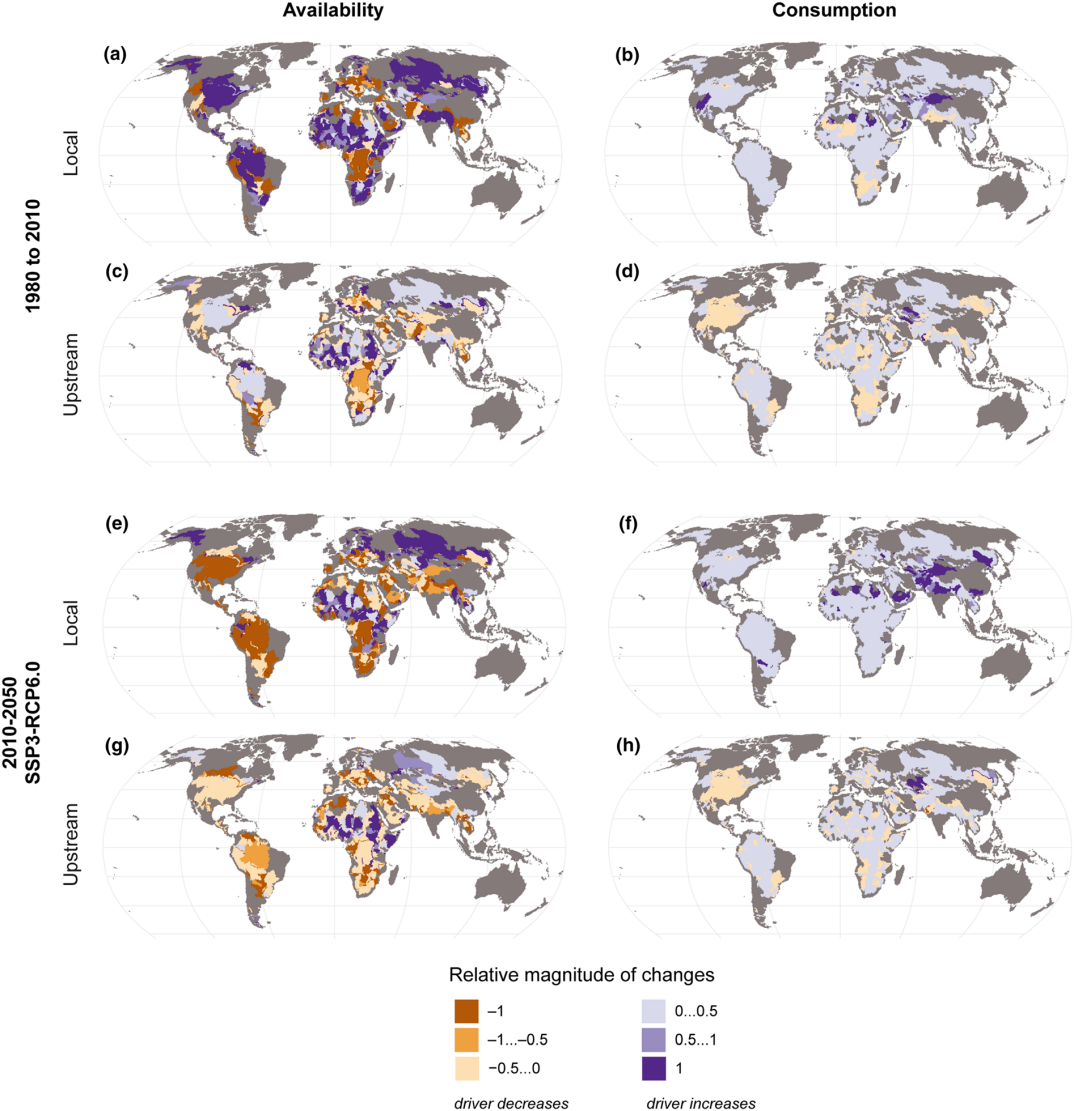
Relative magnitude of changes of local availability, upstream inflows, upstream water consumption, and local water consumption. The result is presented in the scale of −1 to +1, where −1 refers to maximum decrease and +1 refers to maximum increase. For the future, one of the SSP‐RCP scenarios is shown here (see supporting information Figure S5 for other scenarios).

In this section we present the results of relative magnitude of changes of the availability drivers (*LocalWC*, *LocalAA*, *UpstreamWC*, and *UpstreamAA*) to understand how they affect each SBA's water availability (*AA*). Looking at the dominant drivers for changes in a basin's total water availability, this analysis identified that, in almost all scenarios and conditions (both past and future) (Figure 7 and supporting information Figure S5) changes in local and upstream availability are mostly responsible for changes in net water availability rather than changes in upstream consumption. Supporting information Figure S6 shows the drivers of availability that show the most change for the SBAs.

Change in *LocalAA* (local availability)—either decreasing or increasing the water availability—is the largest driver in many parts of the world (Figures 7a and 7e). *LocalWC* (local consumption) is the highest contributing factor mainly in some SBAs in Asia (e.g., Ganges‐Brahmaputra, Tarim), the Middle East (e.g. Saudi Arabia), and northern Africa (e.g., Lake Chad) (Figures 7b and 7f and supporting information Figures S5 and S6). Our results show that change in *UpAA* (upstream availability) is identified as the most important factor for net water availability in some SBAs in Asia, Africa, Europe, North America, and South America for past and all future scenarios, while changes in *UpWC* (upstream consumption) are minor except for very few SBAs in Central Asia (Figures 7d and 7h and supporting information Figures S5 and S6).

#### 4.2.2 Dependency Category

From Table 4, we see an increase in number of SBAs under “hidden dependency” (SNN) condition for all the future scenarios. This means that these subbasins would not experience stress with the used predictions, but potential decreases in upstream inflows would cause stress to occur. Negotiation may therefore be key to preventing this from happening. Also, under scenarios SSP2‐RCP6.0 and SSP3‐RCP6.0, we see an increase in the number of SBAs under “open dependency” (SNS) but a decrease in the population in those SBAs. Open dependency indicates situations where stress occurs in the current situation but would not occur if the upstream water consumption was zero. Stress could be escaped by negotiating reductions in upstream water use as well as reductions in local water use.

**Table 4:** Number of Subbasins (SBAs) and Population Under Different Dependency Categories

Number of subbasins (SBAs) under different dependency categories							
Dependency	Dependency category	1980	2010	2050 SSP1‐RCP2.6	2050 SSP1‐RCP4.5	2050 SSP2‐RCP6.0	2050 SSP3‐RCP6.0
No dependency	NNN	783	770 (−13)	754 (−16)	752 (−18)	747 (−23)	748 (−22)
	SSS	70	79 (+9)	91 (+12)	91 (+12)	94 (+15)	94 (+15)
Hidden dependency	SNN	29	33 (+4)	39 (+6)	39 (+6)	39 (+6)	37 (+4)
Open dependency	SNS	04	4 (0)	2 (−2)	4 (0)	6 (+2)	7 (+3)
Total		886	886	886	886	886	886
Number of people (in millions) under different dependency categories							
No dependency	NNN	1,477	2,322 (+845)	3,067 (+745)	3,061 (+739)	3,322 (+1,000)	3,629 (+1,307)
	SSS	149	302 (+153)	507 (+205)	510 (+208)	585 (+283)	683 (+381)
Hidden dependency	SNN	86	138 (+52)	181 (+95)	177 (+39)	190 (+52)	213 (+75)
Open dependency	SNS	1	32 (+31)	2 (−30)	9 (−23)	27 (−5)	31 (−1)
Total		1,713	2,794	3,757	3,757	4,124	4,556

*Note*. In brackets, we present the changes from the previous year.

The overall analysis shows that in future more SBAs will enter an SSS state, which means more regions will face stress due to high *LocalWC* that cannot be met even with more upstream inflows. No change to upstream consumption could allow stress to be escaped, though it may still be possible to reduce its severity.

## 5 Discussion

In this article, we performed a future scenario analysis to explore the changes in water stress, and prevailing drivers and sources of these changes, in global transboundary basins. Our study provides for the first time a systematic analysis of the impact of different drivers of water scarcity in a transboundary context. This advances the current literature of future water scarcity which concentrates on assessing the changes in stress without addressing these specific drivers (Alcamo et al., 2007; Arnell, 2004; Arnell & Lloyd‐Hughes, 2014; Gosling et al., 2011; Gosling & Arnell, 2016; Oki & Kanae, 2006; Oki et al., 2003; Schewe et al., 2014; Vörösmarty et al., 2000; Wada & Bierkens, 2014). Moreover, our findings greatly contribute to understanding the past and future dynamics in water use and water availability in transboundary river basins in which current literature covers water stress only for present conditions (Degefu et al., 2018, 2019; Munia et al., 2016, 2018; Veldkamp et al., 2017; Wada & Heinrich, 2013). A scenario planning approach is a useful means of coping with predictive uncertainty of both climate and socioeconomic development, particularly for policy‐making purposes (Pegram et al., 2011). The identified drivers of water stress and how they will change in future provide key insights on how to potentially alleviate water stress within basins while taking into consideration the basin‐wide river basin management options. Our study is thus valuable to both academia and policy as a first step assessment to identifying basins where transboundary relationships are strongly affected by changes in socioeconomic and hydro‐climatic variability, and their drivers.

### 5.1 Water Futures: Comparison to Other Studies

Our results suggest that in future water stress will mostly intensify in areas already under stress. Regions where most changes in water stress occur are Central Asia and the northern parts of Africa, whereas in most of Europe and North and South America stress levels do not change notably in any future scenario. We compared our results with the global results on transboundary water scarcity conducted for the current condition. We found that our estimates for population living in water stress areas are somewhat lower compared to the results presented in earlier studies using water consumption (e.g., Degefu et al., 2018, 2019; Munia et al., 2016) and water withdrawal (Munia et al., 2018). One reason is that we used water consumption to estimate water stress, which provides a lower estimate of water stress than using withdrawals—as used in many previous studies. However, water returns (partially) to the river through aquifers and the hydrographic network embedded in the subbasins, so using a withdrawal‐to‐availability ratio provides in many cases an overestimated water stress condition (Veldkamp et al., 2015). Further, estimation of return flows is also uncertain, and the flows may not necessarily be available to downstream users, for example, because of pollution, timing of the flows or infiltration to groundwater (Wada et al., 2011). Difficulty in comparison with other previous studies arises also from the choice of different hydrological models, forcing data, climate, and socioeconomic projections. Our uncertainty analysis (Figure 6) already highlights the significant differences within the used ensemble—which would be further increased when considering other data sources and scenarios. Differences could also come from different geographical units of analysis or different data used for the population distribution.

Our analysis indicates that water stress is and will be dominated by local water consumption rather than upstream consumption or local or upstream availability, for the most part, in both current and future scenarios. The local demand of basins is thus important to manage in order to avoid future stress. This is consistent with findings from previous studies assessing the past development of water scarcity (Kummu et al., 2010; Vörösmarty et al., 2000). Our study explains that this is partly because stress as an indicator places a strong emphasis on local consumption in basins with low water stress (Figure 5), which most subbasins are experiencing (Figure 3). Additionally, consumption tends to vary more than availability does. Water stress could, in most transboundary basins, thus be managed with local water management, supported by negotiations with upstream subbasins.

### 5.2 Model Ensemble Uncertainty

The use of global hydrological models (GHMs)—including their human dimension—has been investigated in a number of (multi‐model) assessments evaluating the impacts of socioeconomic developments and/or hydro‐climatic variability and change on freshwater resources and water scarcity (Haddeland et al., 2014; Prudhomme et al., 2014; Schewe et al., 2014). In this analysis these GHMs are used to specifically evaluate the historical and future development of four drivers of water stress indicators and their impact on water stress condition in a transboundary context. Using multiple estimates of water consumption and availability from ISIMIP and the IIASA WFaS project means that we were able to evaluate the dispersion of water consumption, availability, and stress between global circulation models (GCMs) and GHMs (4 GHMs × 5 GCMs = total of 20 estimates). These dispersion estimates provide information regarding where our findings are more uncertain and where the different model and data combinations agree better among each other. The results arriving from that then highlight areas potentially worthy of investigation due to high or low agreement between different GCMs and GHMs. In general, our analysis shows that the high uncertainty is found in Africa and Central Asia and some parts of Europe for both the water consumption and availability data (Figure 6). The results suggest that specific efforts are needed in reducing variability among different models for estimating the water consumption and availability data for these regions. It should be noted, however, that it was out of scope of this study to investigate the reasons why GCMs and GHMs provide varying values for modeled quantities, including investigation of which water use sector was responsible.

The performance of models has been previously examined in model intercomparison projects (Mueller Schmied et al., 2016; Veldkamp et al., 2018; Wartenburger et al., 2018; Zaherpour et al., 2018). According to these studies, in most part of the world, the models perform rather well against the observations. However, there still exists uncertainties between the models as well as within the models. Further, Veldkamp et al. (2018) found that inclusion of parameterization of human actions (e.g., reservoir operations) in global hydrological models improves generally the estimates of monthly discharge and hydrological extremes, both in managed and near‐natural basins but may lead, however, to overestimate and underestimate in local water availability particularly due to the uncertainties associated with the timing of return flows and reservoir operations. We identified that in the case of GHMs, single models are often responsible for extreme estimates (supporting information Figure S6). This variation between models means that the results are sensitive to the choice of GHM. This explains partly the large spread identified in the analysis, which indicates substantial uncertainties.

### 5.3 Upstream‐Downstream Relations—Implications of Changes in Stress Drivers

Within transboundary river basins, upstream‐downstream asymmetries between countries are often politically sensitive because they offer opportunities for the upstream country to pass on the negative consequences of unsustainable water use to the downstream neighbor (Al‐Faraj & Scholz, 2015; Beck et al., 2014; Drieschova et al., 2008; Veldkamp et al., 2017). Our findings demonstrate that in future, local and upstream availability are expected to significantly influence a basin's net water availability compared to other drivers (local and upstream water consumption). The identified SBAs where maximum changes in upstream availability occur (Figure 4 and supporting information Figure S5) highlight that those regions' availability relies predominantly on actions taken upstream and local decision makers have less control other than to adapt, and naturally do their share to reduce CO_2_ emissions to mitigate climate change. Even though the changes in upstream consumption in future are predicted to have less impact on an SBA's water availability than other drivers (Figure 7) each riparian state's right to have access to a portion of the transboundary water body's water should still be acknowledged, and this situation can be impacted by the negotiations among the riparian states. It would be important to develop management models which help both in reconciling upstream and downstream interests and in increasing social benefits from this critical resource.

Specifically, based on Munia et al.'s (2018) work classifying SBAs according to their upstream dependency, this analysis predicts increases in hidden dependency (SNN) under all the scenarios. This implies a need to maintain good relationships and assess water use and potential changes with upstream basins. Increases in basins falling under SNS and SSS categories indicate cases where institutional arrangements have failed to prevent stress from occurring in future and it may be more worthwhile to look for other solutions, such as those within the political economy literature—concerned with the interaction of political and economic processes in a society, the distribution of power and wealth between different groups and individuals, to create, sustain, and transform these relationships over time (Duncan & Williams, 2012), as discussed by Munia et al. (2018). Understanding when basins become water stressed is particularly important for managers to understand whether they can do something about it themselves, for example, what is the prevailing driver of their basin turning into hidden or open water stress. This may provide insights to various adaptation strategies combating water scarcity.

Poor water management can aggravate the effects of climate change and socioeconomic impact (Kundzewicz et al., 2008), but proper water management can go a long way to offsetting these undesirable impacts. Being sensitive to changes in different drivers of water stress can have substantial implications for the choices being made in the design of adaptation strategies to cope with current and future water scarcity in transboundary basins.

### 5.4 Limitations and Future Work

The results of this analysis clearly depend on the water use and availability scenarios used, and the models and data supporting them. This includes a large number of assumptions, notably using current irrigation areas in future scenarios. The results would most probably be quite different in some basins if irrigation expansion scenarios were adopted (e.g., Hanasaki et al., 2013) or irrigation for bioenergy crops were assumed (e.g., Bonsch et al., 2016), capturing flow‐on and feedback climate change effects more comprehensively. The scenarios used in the analysis are plausible representations of future climate and socioeconomic changes (Wada et al., 2016; Winsemius et al., 2016), and it would be useful to repeat this type of analysis on new scenarios as they emerge.

This analysis uses the water stress indicator to estimate water scarcity, while there also exists other indicators for water scarcity that could be used. Among other findings, our result highlights that water stress is for the most part dominated by local water consumption as by design the indicators places a strong emphasis on local consumption as long as water is sufficiently abundant. Previous research on water scarcity indicators has also criticized this indicator notably for not taking societies' adaptive capacity to cope with stress into account (Damkjaer & Taylor, 2017). Expanding our analysis in future studies to other water scarcity indicators like per capita water availability (shortage) (Falkenmark et al., 2007), food self‐sufficiency (Gerten et al., 2011; Kummu et al., 2014), or sustainability of water withdrawals (Wada et al., 2014) could provide a useful balance to understand more broadly the development of water scarcity in transboundary basins in future, even while this study provides an initial benchmark on which to build.

Environmental protection is an important aspect of management of water resources, notably approached through definition of environmental flow requirements (EFRs). Implementation of environmental flow requirements under climate change is an additional constraint on water allocation, which is not addressed in this analysis. The stress indicator used here does include environmental flow requirements by assuming that 30% of the water is needed to satisfy the EFRs (see, e.g., Falkenmark et al., 2007) but does not account for EFRs in a spatially disaggregated way (Pastor et al., 2014). This should be addressed in more detail, spatially explicitly, in possible future work on the issue.

Since poor water quality has intensified the pressure on water resources (Bayart et al., 2010; van Vliet et al., 2017), it is important to include more specific water quality classes for ecosystem and human uses to enhance the appropriateness of water scarcity assessments. Specifically in case of transboundary basins, industrial or domestic pollution may occur in upstream parts of a basin, which might make water unusable for irrigation or domestic purposes (Thebo et al., 2017). Thus, incorporating water quality is essential for future water scarcity assessments.

In addition, annual assessments of water stress do not account for seasonality and interannual variability of available water. However, using annual averages over a decade provides a conservative estimate of water stress by assuming that water can be stored in sub‐annual scale. To expand our analysis to seasonal or monthly water stress estimates would require accurate information about storage capacities and operations; omitting storage would cause stress to be overestimated. Nevertheless, such analyses have been previously attempted. For example, it has been identified that about 1.6 million people face water scarcity at least for a month of the year for the current situation in transboundary basins (Degefu et al., 2018).

It would also be important to understand how physical water stress in transboundary basins interacts with social aspects when impacting on transboundary relationships. Political power asymmetry along with economic drivers and hydraulic infrastructure development all influence the upstream‐downstream relations (Jägerskog & Zeitoun, 2009). Even though there is a clear evidence of upstream impact on the stress in downstream basins (Degefu et al., 2018, 2019; Munia et al., 2016), critical water disputes mostly come from complex socioeconomic and political interactions (Mirumachi, 2013, 2015). Therefore, calculation of water stress is only one part of the story, which emphasizes the impacts of climate change and human water demand on availability of water resources and pressure on them, while there are naturally many other important factors involved.

## 6 Conclusions

In this paper, we explored the past and future changes in water stress in global transboundary basins using ensemble median results from various global hydrological models and global circulation models. The results of the study indicate that intensification of stress in future scenarios would occur mostly in Central Asia and the northern part of Africa. In most of the subbasin areas (SBAs), the key driver for this intensification is increased local consumption—meaning that if the stressed SBAs, or those approaching to it, want to avoid stress, the single most powerful driver is nearly always local water management. Potential changes in water availability are, nevertheless, important too. Any changes in upstream basins, either due to changing climate or changes in water use, directly impact on downstream water availability—a property that makes transboundary water management challenging. Depending on the dominant driver, different adaptation strategies to cope with the challenge are needed. For example, changes in upstream availability, which was identified as the dominant driver in many basins influencing downstream net water availability in future scenarios, require adaptation strategies beyond local water management. When downstream water availability is influenced by increased upstream demand, negotiations with upstream would be the key strategy. If upstream availability, in turn, would be lowered due to changes in climate, global scale actions on climate change would be the key. In this paper we provide a sample of future scenarios to provide this crucial information for researchers, planners, and other stakeholders to support their work as well as identifying the hot spot areas where more detailed information would be needed.

## Supporting information



Supporting Information S1

## Data Availability

We used data from following sources: ISIMIP portal (https://esg.pik-potsdam.de/search/isimip/), WFaS (Wada et al., 2016), and HYDE population data (Klein Goldewijk et al., 2010; https://themasites.pbl.nl/tridion/en/themasites/hyde/download/index-2.html). The water stress data derived from our analyses is available at http://doi.org/10.5281/zenodo.3898395.
