# Supplementary material for: Future Transboundary Water Stress and Its Drivers Under Climate Change: A Global Study
Source: Earths Future. 2020 Jun 29;8(7):e2019EF001321. doi: 10.1029/2019EF001321 (PMC7380305; doi:10.1029/2019EF001321)
Supplement: Supplementary file 1 — Supporting Information S1 [file EFT2-8-e2019EF001321-s001.pdf]

AGU PUBLICATIONS

Earth's Future

Supporting Information for

Future transboundary water stress and its drivers under climate change: a global study

***Hafsa Ahmed Munia*[1*], *Joseph H.A. Guillaume*[1,2], *Yoshihide Wada*[3,4], *Ted Veldkamp*[3,5], *Vili Virkki*[1], *Matti Kummu*[1*]**

[1]Water and Development Research Group, Aalto University, Finland
[2]The Fenner School of Environment & Society, The Australian National University, Canberra, Australia
[3]International Institute for Applied Systems Analysis, Laxenburg, Austria
[4]Department of Physical Geography, Faculty of Geosciences, Utrecht University, The Netherlands
[5]Institute for Environmental Studies (IVM), Vrije Universiteit Amsterdam, the Netherlands

* corresponding authors: hafsa.munia@aalto.fi, matti.kummu@aalto.fi

Contents of this file

Figures S1 to S8

Introduction

This document provides additional figures done in the analysis-

- Supplementary figure *S1-2, S4-5* represent results for three climate scenarios- SSP1-RCP2.6, SSP1-RCP4.5, and SSP2-RCP6.0.
- Supplementary figure *S3* and *S6-7* represent results for all four climate scenarios used in the analysis.

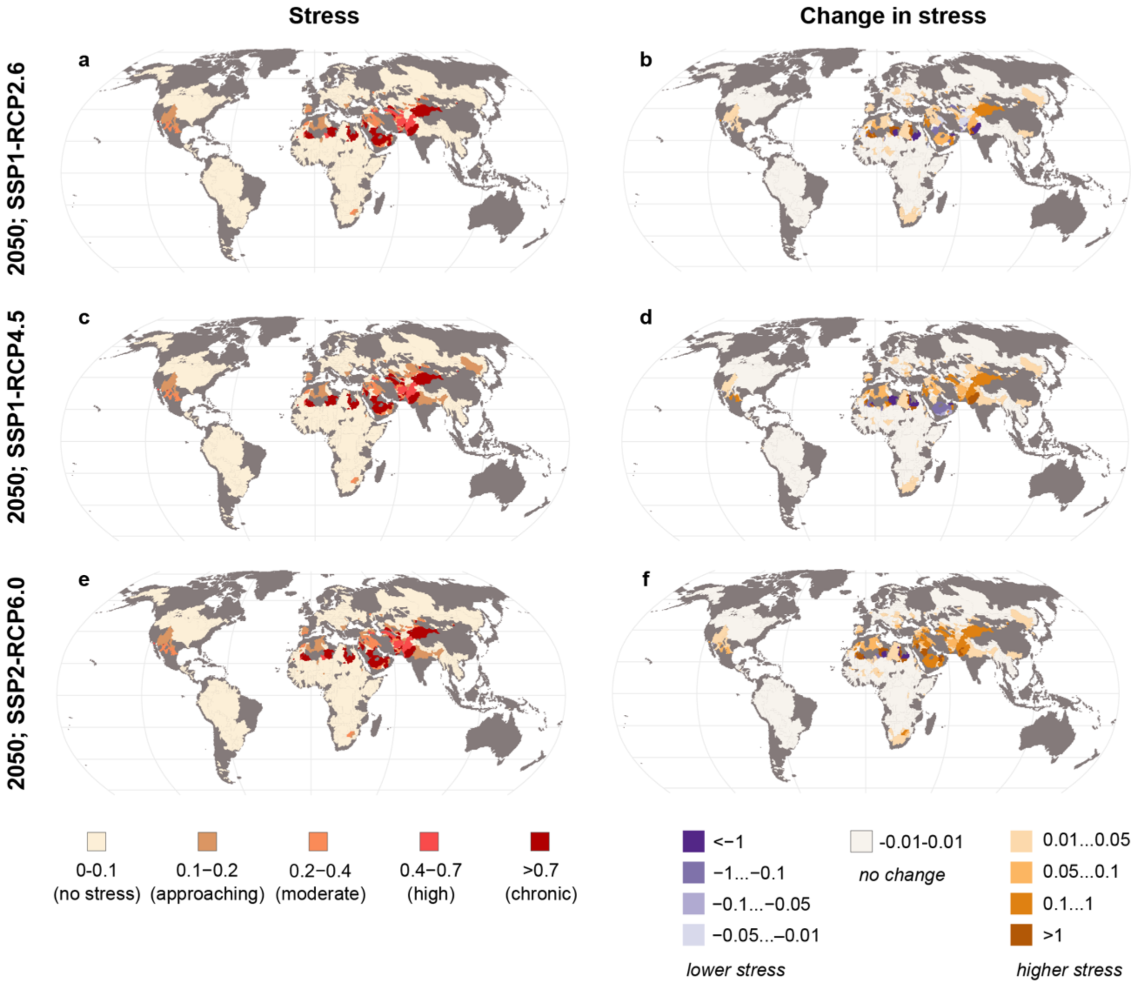

Figure S1. ***Water stress in transboundary sub-basin areas (SBAs) in future conditions (2050) (a, c, e). Changes in stress level from 2010 to 2050 under future scenarios (b, d, e). Negative value means stress level has decreases and positive value means stress level has increases.***

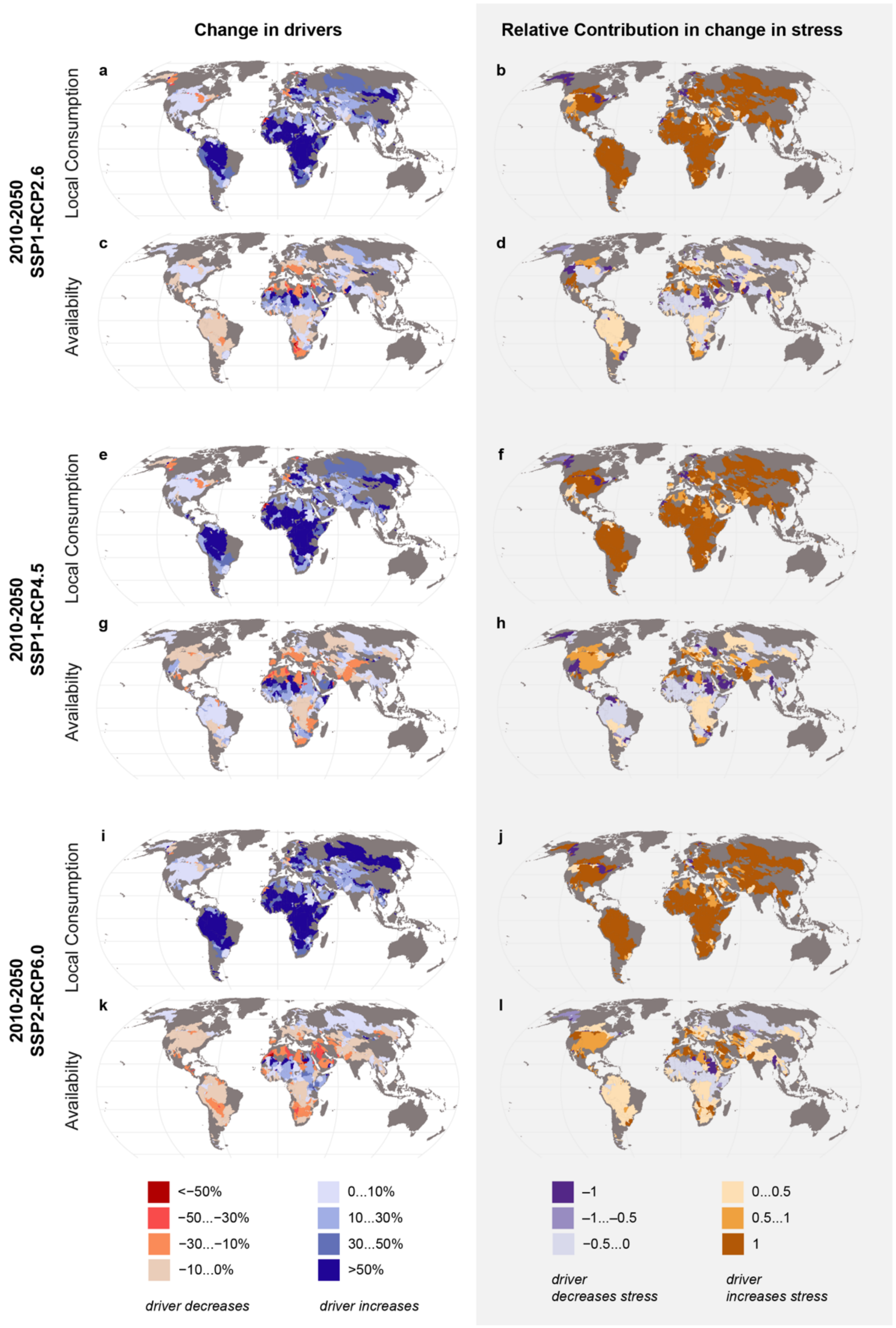

Figure S2. ***Drivers of water stress. Changes in availability and local consumption in 2010 compared to 1980 and in 2050 compared to 2010 under three scenarios (left column). The result is presented as percentage (%) changes. Relative contribution of local consumption (LocalWC) vs availability (AA) to change in stress in 2050 compared to 2010 (right column). The result is presented in the scale of -1 to +1, where -1 refers to maximum contribution in decreasing stress and +1 refers to maximum contribution in increasing stress.***

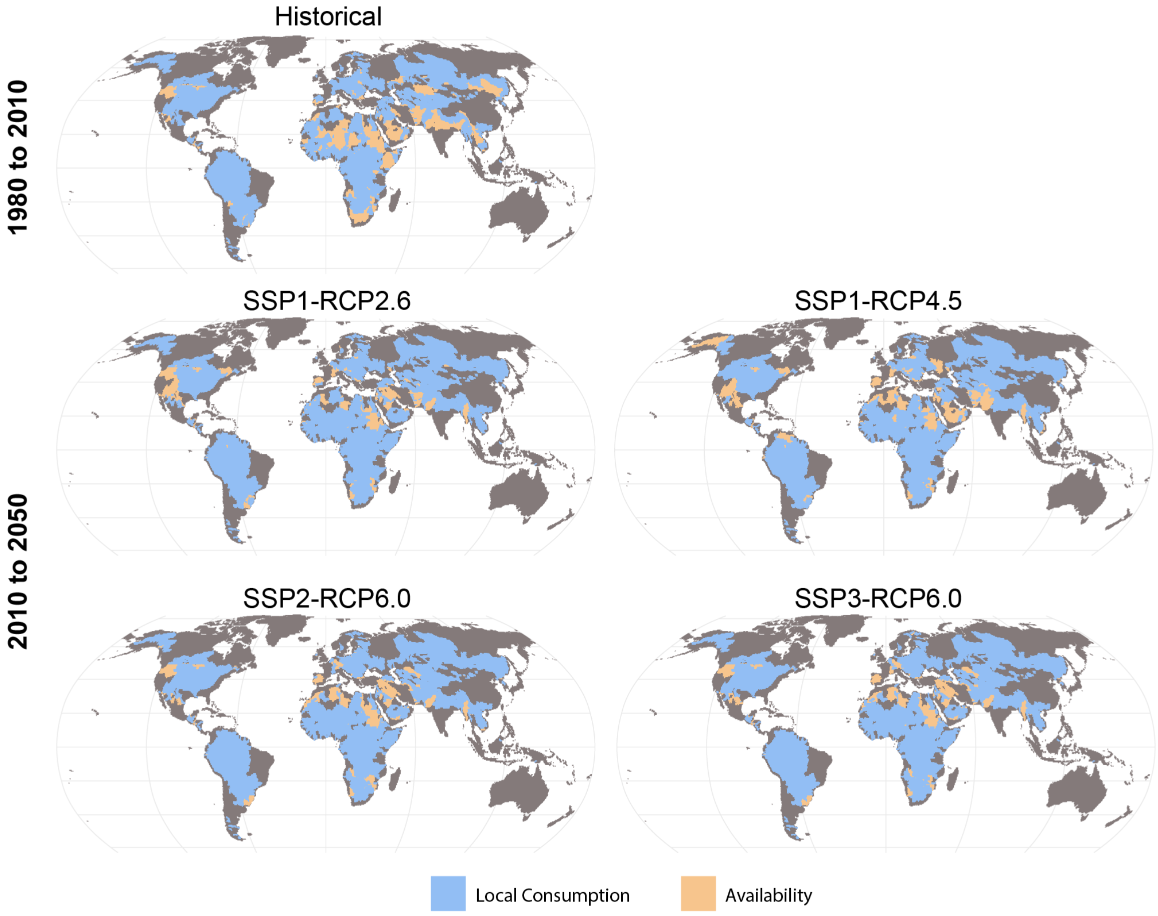

Figure S3. Maximum contribution of local consumption (local WC) vs availability (AA) to change in stress.

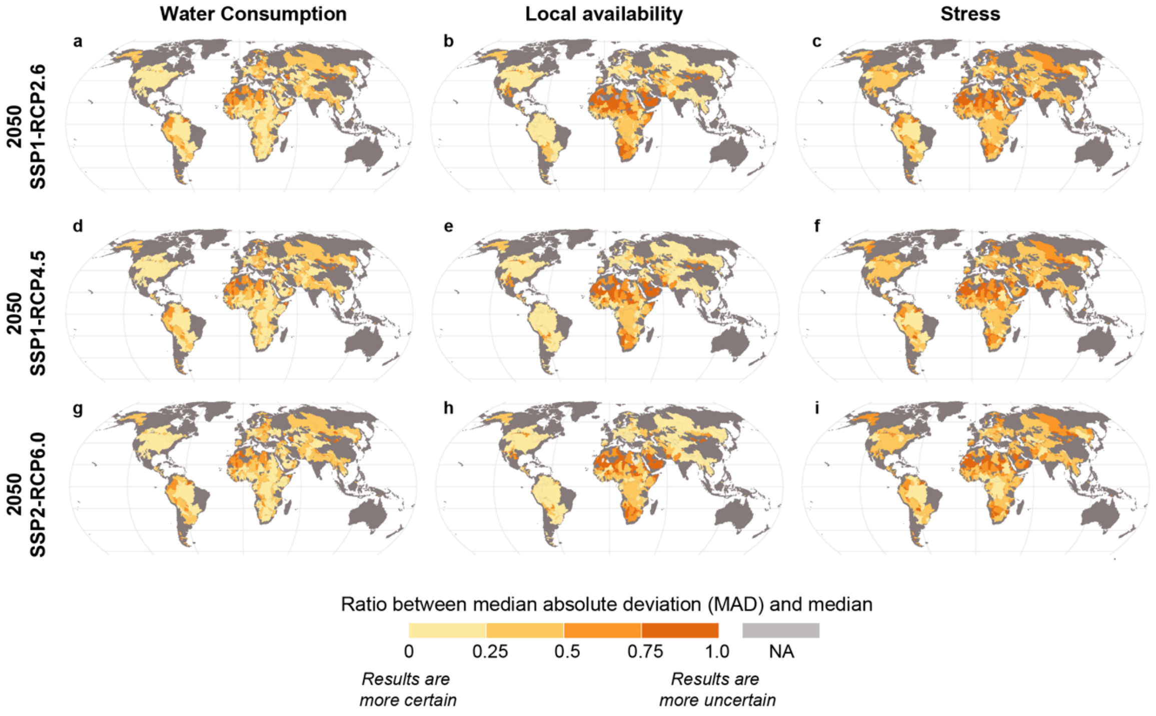

Figure S4. ***MAD/median ratio for local consumption (a, d, g), availability (b, e, h), and stress (c, f, i). NA indicates the median is zero.***

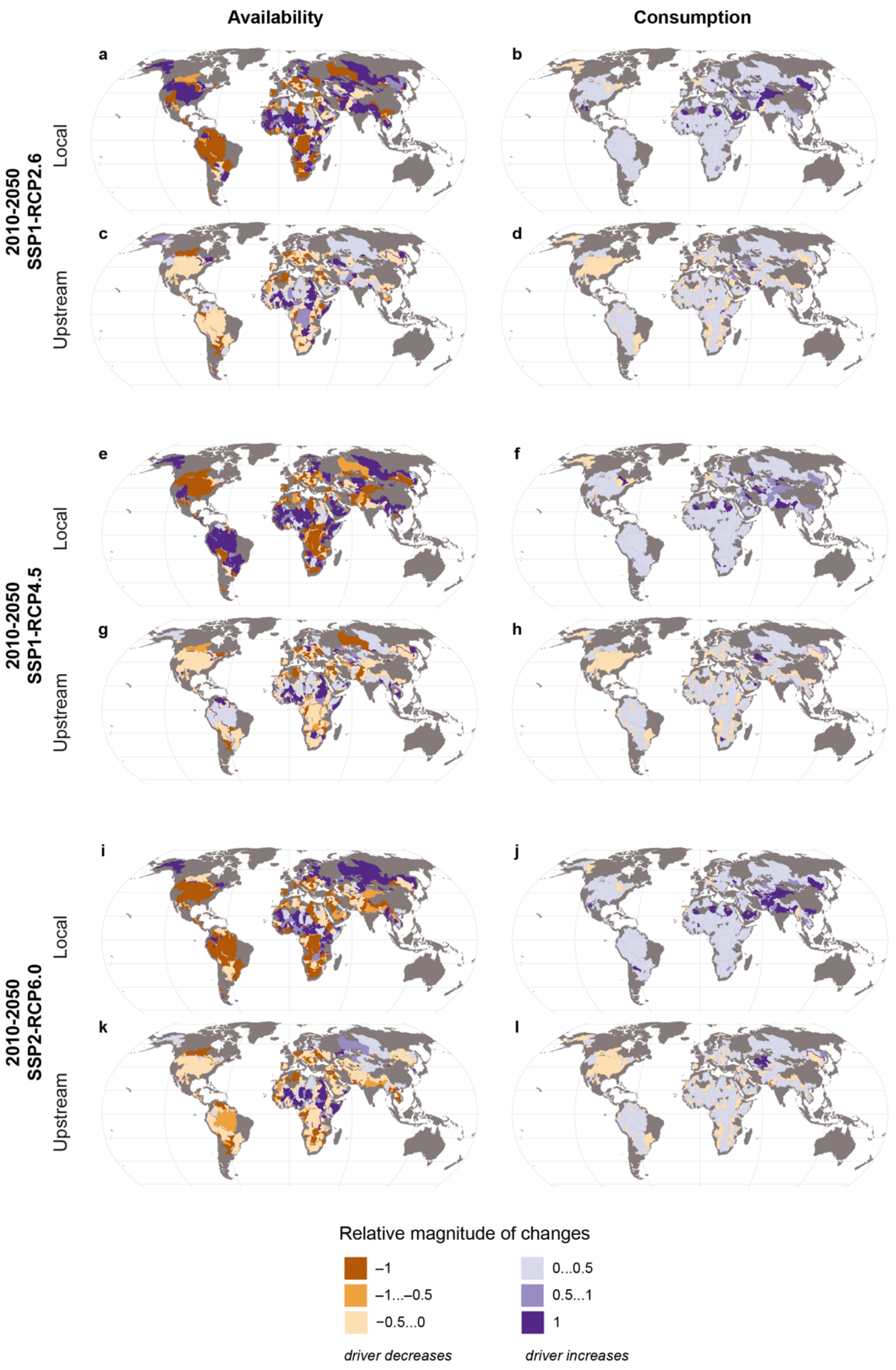

Figure S5. ***Relative magnitude of changes of Local availability, upstream inflows, upstream water consumption and local water consumption. The result is presented in the scale of -1 to +1, where -1 refers to maximum decrease and +1 refers to maximum increase.***

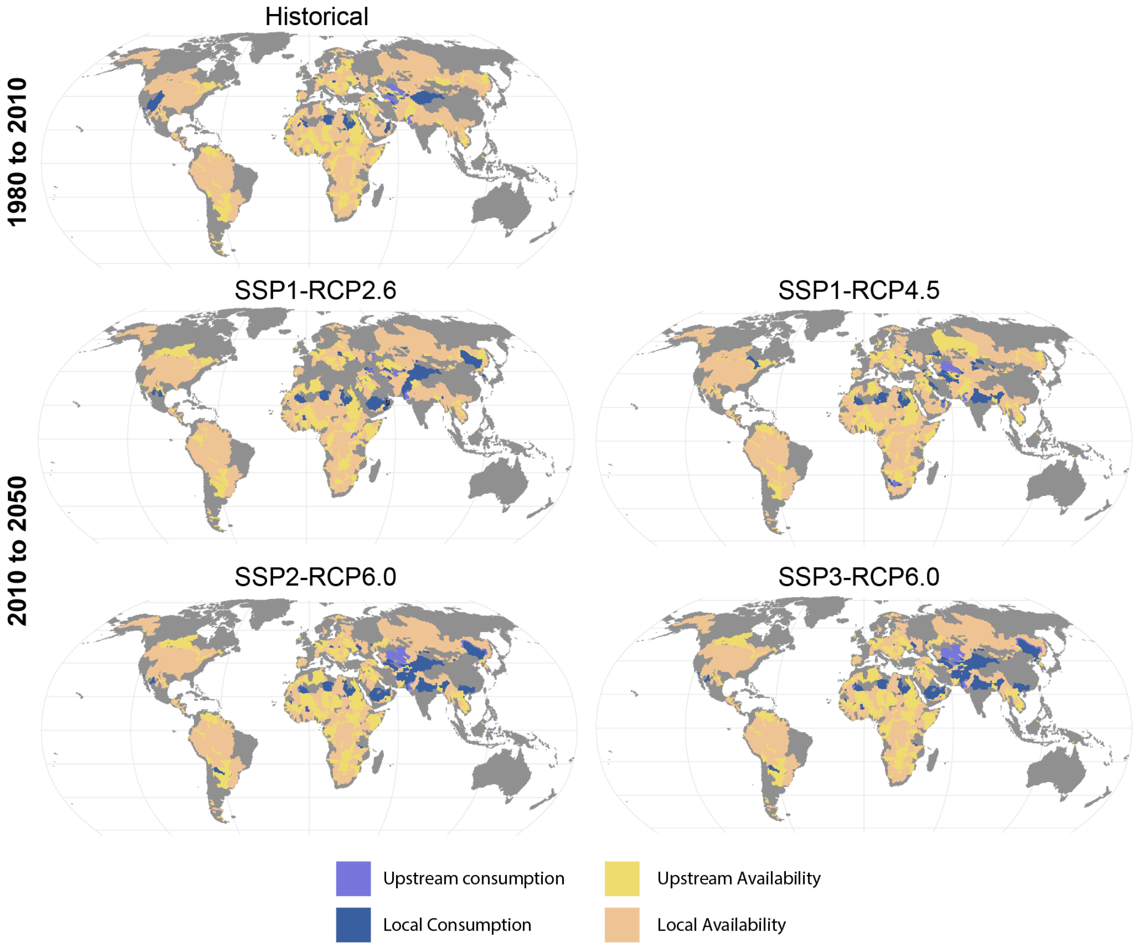

Figure S6. Maximum changing drivers of availability (Local availability, upstream inflows, upstream water consumption and local water consumption).

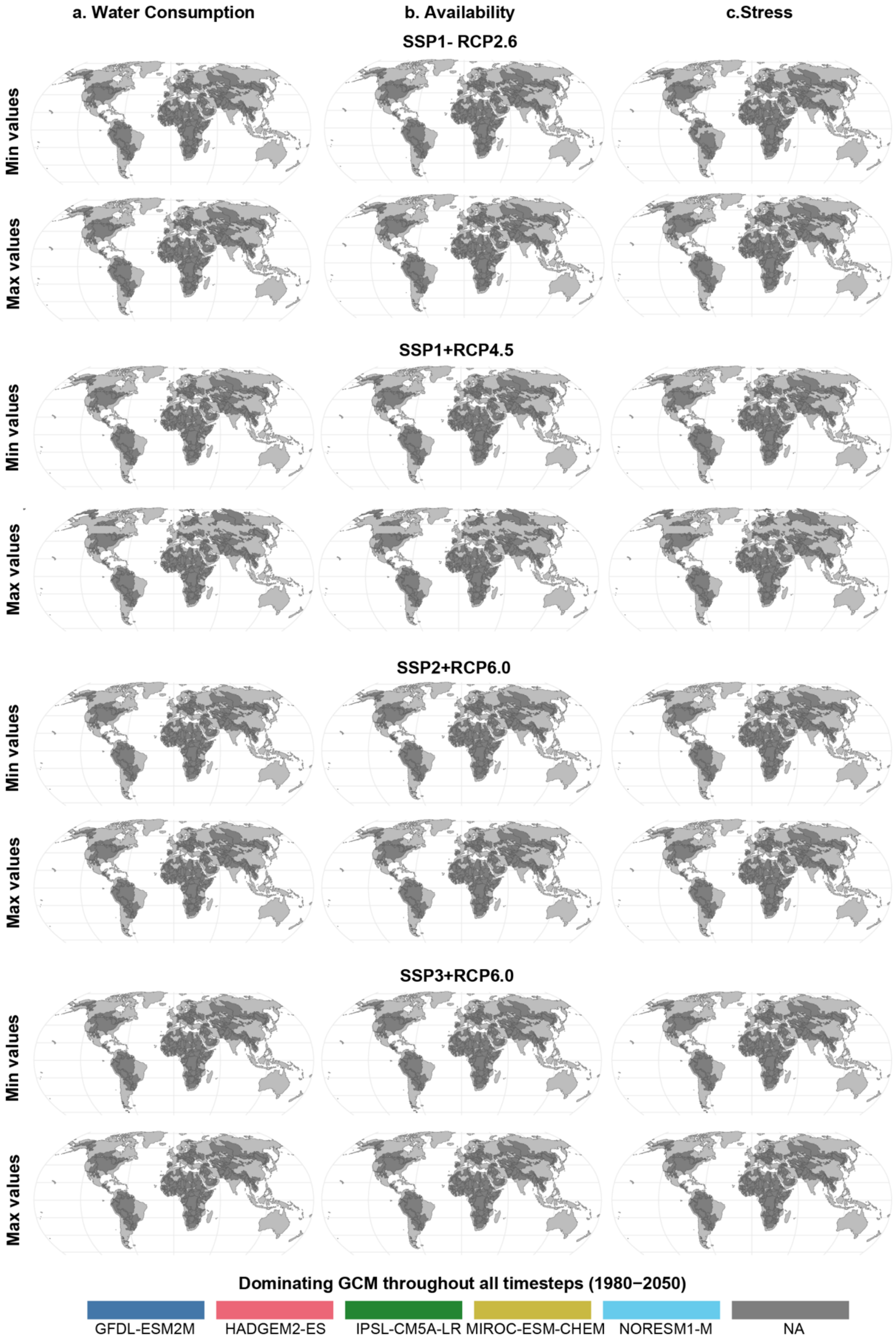

Figure S7. *Dominating Global circulation model (GCM) through all time steps from 1980 to 2050. NA implies while none of the model gives the largest/smallest estimates in every time steps- no specific model domination found.*

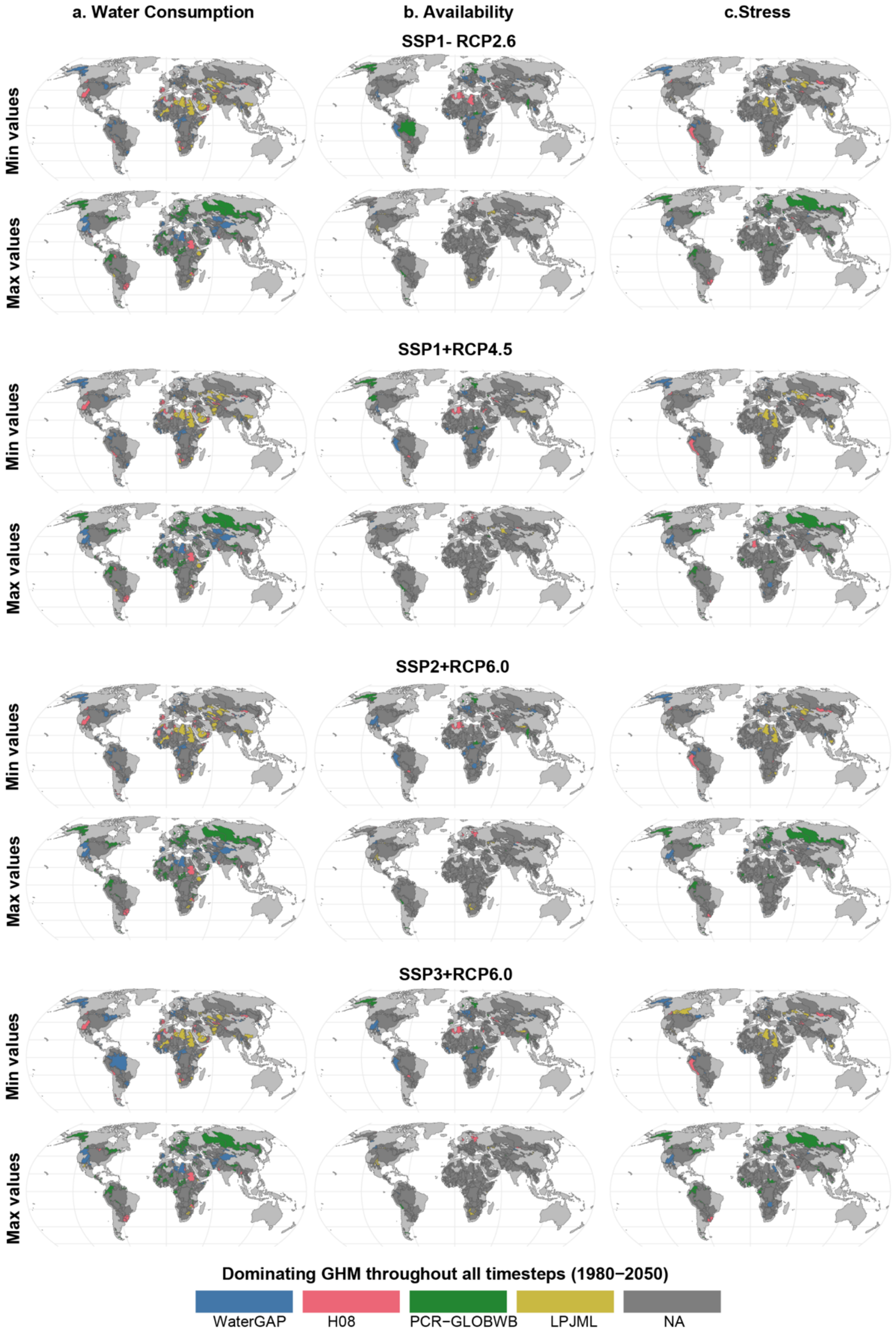

Figure S8. ***Dominating Global hydrological model (GHM) through all time steps from 1980 to 2050. NA implies while none of the model gives the largest/smallest estimates in every time steps-- no specific model domination found.***
